# Molecular Characterization of *ALDH1A1* and Its Association with Secondary Hair Follicle Stem Cell Proliferation in Liaoning Cashmere Goats

**DOI:** 10.3390/cells15201865

**Published:** 2026-10-14

**Authors:** Yuxin Yang, Meijie Wang, Fang Li, Xiaoyi Zhang, Yuxin Cai, Siqi Sun, Wendi Wang, Yixing Fan, Wenlin Bai, Man Bai

**Affiliations:** 1 College of Animal Science and Veterinary Medicine, Shenyang Agricultural University, Shenyang 110866, China; 2025220606@stu.syau.edu.cn (Y.Y.); 18524740625@163.com (M.W.); 2024240759@stu.syau.edu.cn (F.L.); 2025240832@stu.syau.edu.cn (X.Z.); 2025240801@stu.syau.edu.cn (Y.C.); 2026240854@stu.syau.edu.cn (S.S.); 2026220595@stu.syau.edu.cn (W.W.); fanyx@syau.edu.cn (Y.F.);; 2 Engineering Research Center for Animal Molecular Genetics and Breeding of Liaoning Province, Shenyang 110866, China

**Keywords:** *ALDH1A1*, molecular structure, SHFSCs, cell proliferation, Liaoning cashmere goat

## Abstract

**Highlights:**

**What are the main findings?**

• The *ALDH1A1* gene encodes a highly conserved protein and is expressed in multiple tissues of Liaoning cashmere goats, with the highest transcript abundance detected in skin. Its expression in skin was greatest during the seasonally assigned anagen period.
• *ALDH1A1* overexpression increased multiple proliferation-related readouts in primary SHFSCs in vitro, whereas knockdown produced the opposite changes.

**What are the implications of the main findings?**

• *ALDH1A1* may represent an important molecular factor associated with the proliferative activity of SHFSCs and the active growth phase of secondary hair follicles in cashmere goats.
• These findings extend current understanding of secondary hair follicle biology and provide a basis for further investigation of *ALDH1A1* as a candidate molecular target related to cashmere fiber growth.

**Abstract:**

Aldehyde dehydrogenase 1 family member A1 (ALDH1A1) is broadly expressed in mammalian tissues and is a key enzyme in retinoic acid (RA) biosynthesis, a process closely linked to hair follicle regeneration. Its expression pattern and function in cashmere goats, however, remain poorly defined. In this study, we isolated and characterized the full-length open reading frame (ORF) of *ALDH1A1* cDNA from Liaoning cashmere goat skin, examined its expression across multiple tissues and seasonally assigned hair-cycle periods, and evaluated its effects on the proliferation of secondary hair follicle stem cells (SHFSCs). Nine male goats were used for seasonal skin sampling (*n* = 3 per period), primary SHFSCs were derived from one additional female donor (*n* = 1), and cell-based assays were independently repeated three times. The *ALDH1A1* ORF was 1506 bp and encoded a 501-amino-acid protein; its nucleotide sequence shared 90.07–99.14% identity with corresponding mammalian sequences. Conserved-domain analysis assigned the goat ALDH1A1 protein to the aldehyde dehydrogenase superfamily (ALDH-SF), and phylogenetic analysis showed that the experimentally obtained sequence clustered with the *Capra hircus* reference sequence and other ruminant ALDH1A1 sequences. Among the tissues examined, *ALDH1A1* transcripts were most abundant in skin, and both mRNA and protein levels in skin were highest during the seasonally assigned anagen period. In SHFSCs, *ALDH1A1* overexpression increased multiple proliferation-related readouts, whereas knockdown produced the opposite changes. Overall, these findings characterize goat ALDH1A1 at the molecular and functional levels and support an association between *ALDH1A1* perturbation and proliferation-related phenotypes in this primary-cell model; however, they do not establish whether these effects are mediated by RA-dependent signaling.

## 1. Introduction

Cashmere is the principal high-value fiber produced by cashmere goats and is prized for its fineness, softness, thermal insulation, moisture absorption, and breathability; for these properties, it is often described as “soft gold” [1]. Goat fleece contains both wool and cashmere fibers [2], which arise mainly from primary and secondary hair follicles, respectively [3]. Primary follicles mainly form protective wool, whereas secondary follicles generate the fine cashmere fibers that provide thermal insulation [4]. Secondary follicles show a pronounced annual cycle, while primary follicles are comparatively stable [5]. The lengths of anagen, catagen, and telogen vary among cashmere goat breeds [6,7,8]. In Liaoning cashmere goats, anagen occurs from April to November, catagen from December to January, and telogen from February to March, with corresponding changes in follicular architecture, cellular state, and gene expression [9]. Hair follicle stem cells (HFSCs) drive follicular renewal through stage-dependent shifts among quiescence, activation, proliferation, and differentiation [10,11]. Changes in the local niche can activate otherwise quiescent HFSCs and initiate proliferative and differentiation responses involved in follicular regeneration and skin repair [12,13]. Environmental cues such as temperature, humidity, and photoperiod, together with coordinated changes in follicle-related genes, proteins, and signaling pathways, also influence secondary follicle cycling [14,15,16,17,18,19,20]. Identifying genes associated with HFSC activity is therefore important for clarifying how the secondary follicle cycle is regulated.

Aldehyde dehydrogenase 1 family member A1 (ALDH1A1) is a retinaldehyde dehydrogenase of the aldehyde dehydrogenase (ALDH) superfamily and is broadly expressed across mammalian tissues. Previous work has associated *ALDH1A1* with stem-cell properties, tumor biology, and tissue development [21,22,23]. By oxidizing retinaldehyde to retinoic acid (RA), ALDH1A1 contributes to RA homeostasis. Appropriate RA homeostasis is important for normal hair follicle morphogenesis and differentiation, whereas its disruption can lead to abnormal epidermal and follicular keratinization and impaired hair growth [22,24]. In mice, RA treatment has been reported to promote the transition of hair follicles from the resting state toward active growth together with increased HFSC proliferation [25], and Wnt/β-catenin signaling has been implicated in RA-associated HFSC activation [26]. Because *ALDH1A1* contributes to RA biosynthesis, these observations raise the hypothesis that *ALDH1A1* may participate in hair follicle cycling and HFSC function [27]. However, whether any effect of *ALDH1A1* on cashmere goat secondary hair follicle stem cells (SHFSCs) is mediated by RA-dependent signaling remains unknown. *ALDH1A1* expression is enriched during anagen in mouse hair follicles [28], and *ALDH1A1* has also been proposed as a candidate gene for cashmere goat HFSC proliferation [29]. These findings provided a rationale to examine whether *ALDH1A1* is involved in the proliferative activity of cashmere goat SHFSCs. Nevertheless, its molecular features, expression pattern, and function in these cells remain insufficiently characterized, as most previous *ALDH1A1* studies have focused on tumor systems.

Accordingly, we cloned and characterized the full-length open reading frame (ORF) of *ALDH1A1* cDNA from Liaoning cashmere goat skin, quantified *ALDH1A1* expression in multiple tissues and across seasonally assigned hair-cycle periods, and tested the effect of *ALDH1A1* on SHFSC proliferation. This study adds molecular and functional information for goat *ALDH1A1* and helps define its relationship with SHFSC proliferation in a primary cell model.

## 2. Materials and Methods

### 2.1. Ethical Statement

The experimental procedures were approved by the Laboratory Animal Welfare and Ethics Review Committee of Shenyang Agricultural University (Ethical Code: No. SNLL25030707), and all animal-related procedures were carried out in accordance with the approved protocol.

### 2.2. Animal Sampling and Tissue Collection

Nine healthy adult male Liaoning cashmere goats aged 2–3 years and weighing approximately 70–80 kg were obtained from the nucleus breeding herd of the Liaoning Provincial Academy of Animal Husbandry Science. All animals were maintained under the same husbandry conditions with natural light and free access to feed and water. According to the established seasonal hair-cycle pattern reported for Liaoning cashmere goats [9], three independent goats were sampled at each of three time points, September, December, and March, corresponding to the expected anagen, catagen, and telogen periods, respectively. Skin biopsy was performed under local anesthesia using multipoint subcutaneous infiltration of 2% lidocaine (Sigma-Aldrich, St. Louis, MO, USA) at a total dose not exceeding 7 mg/kg. After 5–10 min, adequate anesthesia was confirmed by the absence of a pain response to needle-prick stimulation. One 2 cm × 2 cm skin biopsy was collected from the scapular region of each goat. The incision was closed with interrupted absorbable sutures, disinfected with povidone–iodine (Sigma-Aldrich, St. Louis, MO, USA), and covered with a sterile dressing. Meloxicam (0.5 mg/kg, intramuscularly; Sigma-Aldrich, St. Louis, MO, USA) was administered immediately after biopsy and once daily for the following two days. No systemic prophylactic antibiotics were used. Animals were monitored once daily for three consecutive days for local redness or exudation, feed intake, general condition, spontaneous activity, and pain-related behavior. Each goat underwent skin sampling only once. Hair-cycle stages were assigned on the basis of the documented seasonal pattern, and histological confirmation was not available for these samples.

The three goats sampled during the telogen period were electrically stunned, and loss of consciousness was confirmed before carotid exsanguination. Heart, liver, spleen, lung, kidney, and testis tissues were collected immediately thereafter. Together with the corresponding telogen skin samples from the same animals, these samples comprised the seven tissues examined in this study. All collected tissues were immediately frozen in liquid nitrogen and stored at −80 °C until subsequent analysis. No a priori power calculation was performed; the animal sample size was selected with reference to comparable tissue-expression studies in cashmere goats.

### 2.3. Isolation and Culture of Primary SHFSCs

Primary SHFSCs used in this study were isolated from skin in the scapular region of a separate female Liaoning cashmere goat aged 3 years (single donor, *n* = 1). Skin tissue was washed with sterile phosphate-buffered saline (PBS), the connective tissue was removed, and the tissue was minced and digested with 0.25% neutral protease (Roche, Basel, Switzerland) at 4 °C for 8 h. Under a stereomicroscope (Olympus, Tokyo, Japan), secondary hair follicles (SHFs) were mechanically isolated using a sterile scalpel and disposable syringe needle and subsequently digested with 0.2% type II collagenase (Roche, Basel, Switzerland) for 30 min. Digestion was terminated with F-12 medium (Gibco, Grand Island, NY, USA) containing 10% fetal bovine serum (FBS; Gibco, Grand Island, NY, USA), after which the cell suspension was passed through a 100-mesh cell strainer and centrifuged (Eppendorf, Hamburg, Germany) at 1000 rpm for 5 min. The resulting cell pellet was resuspended in F-12 medium supplemented with 10% FBS and 1% penicillin–streptomycin (Gibco, Grand Island, NY, USA) and cultured at 37 °C with 5% CO_2_ in a humidified incubator (Thermo Fisher Scientific, Waltham, MA, USA); the medium was replaced daily [30,31]. At approximately 80% confluence, P1 cells were dissociated with 0.25% trypsin (Gibco, Grand Island, NY, USA) and either continuously passaged or cryopreserved in liquid nitrogen using a freezing medium containing 10% dimethyl sulfoxide (DMSO; Sigma-Aldrich, St. Louis, MO, USA) and 90% FBS. Depending on the experiment, cells were either continuously passaged from the freshly isolated culture or used after one or two cryopreservation–recovery cycles. Cryopreserved cells were stored in liquid nitrogen for approximately six months; after recovery, the cells were passaged once and used when they again reached approximately 80% confluence. No fixed culture duration was applied; cells were used according to passage number and after reaching approximately 80% confluence. Mycoplasma contamination was assessed by Polymerase chain reaction (PCR), and only cultures that tested negative were used for experiments. All functional assays were performed using cells at passages 2–4 (P2–P4).

### 2.4. Immunofluorescence Characterization of SHFSCs

For phenotypic characterization of the cultured SHFSCs, passage 1 (P1) cells were subjected to immunofluorescence staining for KRT15 and KRT19. Cells were seeded onto sterile glass coverslips in 24-well plates at 2 × 10^4^ cells per well in 1 mL of culture medium and allowed to adhere before staining. Cells were washed with PBS, fixed with 4% paraformaldehyde (Sigma-Aldrich, St. Louis, MO, USA) at 4 °C for 30 min, and washed three times with PBS for 5 min each. Cells were then permeabilized and blocked for 2 h using a Triton X-100-containing blocking solution supplemented with 10% serum (Sigma-Aldrich, St. Louis, MO, USA). Coverslips were incubated overnight at 4 °C with primary antibodies against KRT15 and KRT19, followed by the corresponding fluorescent secondary antibodies for 2 h at room temperature in the dark. After washing, nuclei were counterstained with 4′,6-Diamidino-2-phenylindole (DAPI) (1:1000) for 5 min. Coverslips were mounted with Fluoromount-G (Southern Biotech, Birmingham, AL, USA) and examined using an Olympus IX73 fluorescence microscope (Olympus, Tokyo, Japan). Negative controls were processed identically except for the omission of the primary antibody.

### 2.5. RNA Extraction, cDNA Synthesis, and Primer Design

Total RNA was extracted from collected tissues and cultured SHFSCs using FreeZol Reagent (Vazyme, Nanjing, China). cDNA was subsequently synthesized using the PrimeScript FAST RT Reagent Kit with gDNA Eraser (Takara, Dalian, China) according to the manufacturer’s instructions.

The *Capra hircus ALDH1A1* reference sequence (XM_005683753.3) was obtained from NCBI GenBank. Primer Premier 6.0 was used to design gene-specific primers for amplification of the *ALDH1A1* cDNA ORF and for expression analysis. Additional expression-analysis primers targeted *KRT19*, *ITGB1*, *PCNA*, and *β-actin*, with *β-actin* used as the reference gene. Primer sequences are provided in Supplementary Table S1.

### 2.6. Cloning of the ORF of ALDH1A1 cDNA

The Liaoning cashmere goat *ALDH1A1* ORF was amplified from cDNA by PCR using Premix Taq (Takara, Dalian, China) in a 50 μL reaction. The cycling program was 94 °C for 120 s, followed by 34 cycles of 94 °C for 30 s, 57 °C for 30 s, and 72 °C for 90 s, with a final extension at 72 °C for 2 min. Amplification products were separated on a 1% agarose gel, and the target bands were excised and submitted to Sangon Biotech Co., Ltd. (Shanghai, China) for sequencing. The experimentally obtained *ALDH1A1* ORF sequence was deposited in GenBank under accession number PZ619936.

### 2.7. Sequence Analysis of ALDH1A1

Sequence assembly, alignment, and translation of the *ALDH1A1* ORF were performed in DNAMAN 9.0 under default settings. The experimentally obtained *ALDH1A1* ORF sequence was aligned with the *Capra hircus* reference transcript XM_005683753.3 to identify nucleotide sequence differences. Because independent high-fidelity re-amplification and genomic DNA validation were not performed, these differences were treated descriptively as sequence differences relative to the reference transcript. For phylogenetic analysis, representative ALDH1A1 amino acid sequences were aligned using MUSCLE in MEGA 11. A neighbor-joining tree was constructed using p-distance, with pairwise deletion of gaps and missing data and 1000 bootstrap replicates. The tree was rooted using ALDH1A2 as the outgroup, and branch lengths were expressed as p-distance. ExPASy ProtParam was used to determine basic physicochemical characteristics of the deduced ALDH1A1 protein. SignalP 6.0 and TMHMM 2.0 were used to predict signal peptide and transmembrane helices, respectively, and NetPhos 3.1 was used to identify potential phosphorylation sites. Conserved regions were annotated using the NCBI Conserved Domain Database (CDD), and secondary-structure composition was estimated using SOPMA. Homology modeling was performed using SWISS-MODEL, with the optimal template selected automatically by the server. Model quality was evaluated using the SWISS-MODEL quality estimates, ProSA-web, and MolProbity v4.4. PyMOL 3.0 was used for three-dimensional visualization and annotation of conserved structural regions and catalytic residues. Access details for the software, databases, and online tools are provided in Supplementary Table S2.

### 2.8. Construction of the ALDH1A1 Overexpression Vector and RNA Interference

To generate the overexpression construct, the complete goat *ALDH1A1* coding sequence was amplified using Premix Taq (Takara, Dalian, China) and inserted into the pcDNA3.1(+) vector (Atantares, Suzhou, China) through the KpnI and EcoRI restriction sites. Because neither restriction site occurs within the *ALDH1A1* coding region, the KpnI (GGTACC) and EcoRI (GAATTC) recognition sequences were incorporated into the cloning primers. The recombinant plasmid was screened by restriction digestion and further verified by Sanger sequencing of the complete *ALDH1A1* insert and the flanking vector–insert regions before use. Before the functional experiments, transfection conditions were optimized in 6-well plates using pcDNA3.1(+)-*ALDH1A1* at 0.1, 0.3, 0.5, 1.0, and 2.0 μg/well (*ALDH1A1* overexpression, OE-*ALDH1A1*). *ALDH1A1* overexpression efficiency was evaluated by Quantitative real-time PCR (qRT-PCR) and Western blotting. Cells transfected with the corresponding amount of empty pcDNA3.1(+) vector served as the overexpression control (OE-control).

For gene knockdown, three small interfering RNAs (siRNAs) targeting goat *ALDH1A1* and a non-targeting control siRNA (si-control) were designed (Atantares, Suzhou, China; Supplementary Table S3). Before the functional experiments, transfection conditions were optimized in 6-well plates by evaluating final siRNA concentrations of 50, 75, 100, and 200 nM. The three *ALDH1A1*-targeting siRNAs (si-*ALDH1A1*-1, si-*ALDH1A1*-2, and si-*ALDH1A1*-3) were evaluated, and *ALDH1A1* knockdown efficiency was assessed by qRT-PCR and Western blotting. Cells transfected with the non-targeting siRNA served as the knockdown control (si-control).

During transfection optimization, morphological tolerability was assessed by bright-field microscopy. A transfection condition was considered morphologically acceptable when cells retained their typical adherent morphology and attachment, without an obvious increase in detached or floating cells relative to the corresponding control. Conditions that produced reproducible changes in *ALDH1A1* expression while meeting these morphological criteria were considered suitable for subsequent functional experiments. Transfection was performed using the Lipofectamine 3000 Transfection Reagent system (Invitrogen, Carlsbad, CA, USA) under conditions established in preliminary optimization experiments. Reagent-only controls receiving the corresponding transfection reagents without plasmid or siRNA were included during transfection optimization. Transfection efficiency was evaluated 48 h after transfection.

### 2.9. CCK-8 Metabolic Activity Assay

Cellular metabolic activity was assessed using the Cell Counting Kit-8 (CCK-8) assay. Four parallel 96-well plates were prepared in each independent experiment, with one plate assigned to each time point (0, 24, 48, or 72 h). Each plate contained the four treatment groups with five technical replicate wells per group. At the designated time point, 10 μL of CCK-8 reagent (Vazyme, Nanjing, China) was added to each well, followed by incubation for 2 h at 37 °C in the dark, and absorbance at 450 nm was recorded using a microplate reader (BioTek Instruments, Winooski, VT, USA). Each plate was used for a single time-point measurement and was not returned to culture thereafter. The five technical replicate wells were averaged within each independent experiment, and the assay was independently repeated three times. Absorbance values were corrected by subtracting the corresponding blank-well absorbance before analysis. The blank-corrected OD450 values were used directly for graphical presentation and statistical analysis without further normalization. The independent CCK-8 validation using si-*ALDH1A1*-2 and its corresponding si-control followed the same procedure and observation time points.

### 2.10. EdU Assay

DNA synthesis was measured using a 5-ethynyl-2′-deoxyuridine (EdU) Cell Proliferation Assay Kit (Beyotime, Shanghai, China). Following transfection, SHFSCs were incubated with 10 μM EdU for 2 h and then fixed and permeabilized before the Click reaction and counterstained with DAPI, according to the manufacturer’s instructions. Three technical replicate wells were included for each group, and the assay was independently repeated three times. Fluorescence images were acquired using an inverted microscope (Olympus IX73, Tokyo, Japan) under identical exposure settings for all groups. Five non-overlapping fields were randomly selected from each well, excluding well-edge regions and areas with obvious cell aggregation. DAPI staining was used to identify total nuclei. The fluorescence-intensity threshold for EdU positivity was established using EdU-negative control samples and was applied consistently across treatment groups. Nuclei showing EdU fluorescence above the background threshold were classified as EdU-positive. All nuclei within each selected field were included in the analysis. Quantification was performed in ImageJ 1.54m by an investigator blinded to treatment allocation, and the EdU-positive fraction was calculated as the number of EdU-positive nuclei divided by the total number of DAPI-stained nuclei.

### 2.11. Cell-Cycle Analysis

Forty-eight hours after transfection, approximately 1 × 10^6^ SHFSCs were collected from each sample and fixed overnight in absolute ethanol pre-cooled to −20 °C. After washing with PBS, cells were treated with RNase A at a final concentration of 100 μg/mL and stained with propidium iodide (PI; Thermo Fisher Scientific, Waltham, MA, USA) at a final concentration of 50 μg/mL. Samples were analyzed using a BD FACSCelesta flow cytometer (BD Biosciences, Franklin Lakes, NJ, USA) with BD FACSDiva Software v9.0. The main cell population was first identified on forward-scatter area (FSC-A) versus side-scatter area (SSC-A) plots to exclude cellular debris, followed by singlet selection using PI-A versus PI-W to exclude doublets and cell aggregates. At least 10,000 singlet events were acquired for each sample. DNA-content distributions within the singlet population were then analyzed in FlowJo v10.8 using the Watson pragmatic model to estimate the proportions of cells in the G0/G1, S, and G2/M phases. Within each independent experiment, the corresponding control and treatment samples were fixed, stained, and analyzed in parallel. Cell-cycle experiments were independently repeated three times.

### 2.12. Colony-Formation Assays

For colony formation, transfected SHFSCs were seeded in 6-well plates at 1000 cells per well. Cells were cultured for 5–7 days, with the assay terminated when discrete colonies with clearly distinguishable boundaries had formed but before substantial fusion between neighboring colonies occurred. An independent colony was defined as a spatially separated cell cluster containing at least 50 cells; substantially fused colonies were excluded from analysis. Three technical replicate wells were included for each treatment, and the assay was independently repeated three times. Colonies were fixed with 4% paraformaldehyde, stained with 0.1% crystal violet, and imaged. Colony counting was performed by an investigator blinded to treatment allocation. Plating efficiency was calculated as the number of colonies formed divided by the number of cells seeded and multiplied by 100%.

### 2.13. Quantitative Real-Time PCR

RNA isolation and cDNA synthesis followed the procedures described in Section 2.5. qRT-PCR was carried out using a CFX96 Real-Time PCR System (Bio-Rad, Hercules, CA, USA) and ChamQ Universal SYBR qPCR Master Mix (Vazyme, Nanjing, China), with *β-actin* as the reference gene. The thermal program consisted of 95 °C for 30 s followed by 40 cycles of 95 °C for 10 s and 60 °C for 20 s. Melting-curve analysis was performed from 65 °C to 95 °C in 0.5 °C increments every 10 s, and a negative control was included in each run.

Relative transcript abundance was calculated using the 2^−ΔΔCt^ method. For cell-based perturbation experiments, *ALDH1A1*, *KRT19*, *ITGB1*, and *PCNA* transcript levels were quantified 48 h after transfection. *KRT19* and *ITGB1* were included as SHFSC-associated molecular markers, whereas *PCNA* was included as a proliferation-associated readout. For the seven-tissue expression analysis, the same three telogen goats served as biological replicates (*n* = 3). For the hair-cycle expression analysis, three independent goats were analyzed at each seasonally assigned period (*n* = 3 per period; nine goats in total). Cell-based qRT-PCR experiments were independently repeated three times using the same donor-derived SHFSC preparation. All qRT-PCR measurements were performed with three technical replicates per sample.

### 2.14. Western Blotting

Total protein was extracted from tissue and cell samples with a Total Protein Extraction Kit (Yase, Shanghai, China), and protein concentration was determined by the bicinchoninic acid (BCA) assay (Yase, Shanghai, China). For ALDH1A1 detection, 50 μg of protein was loaded per lane because endogenous ALDH1A1 abundance was relatively low; 30 μg per lane was used for KRT19, integrin β1, and PCNA because this amount produced clear and reproducible bands. Equal amounts of total protein were loaded across samples within each blot. Glyceraldehyde-3-phosphate dehydrogenase (GAPDH) detected on the corresponding membrane or membrane section served as the loading control. Depending on the target and molecular-weight range, membranes were either cut prior to antibody incubation or sequentially stripped and reprobed. After sodium dodecyl sulfate–polyacrylamide gel electrophoresis (SDS-PAGE), proteins were transferred to polyvinylidene difluoride (PVDF; Millipore, Burlington, MA, USA) membranes with a rapid transfer system for 9 min. The membranes were blocked for 10 min with protein-free blocking buffer and washed three times with Tris-buffered saline containing Tween 20 (TBST), 10 min per wash. Primary antibodies were incubated overnight at 4 °C with gentle agitation, followed by horseradish peroxidase (HRP)-conjugated secondary antibodies for 30 min at 37 °C with agitation. Antibody sources and working dilutions are listed in Supplementary Table S4. Signals were developed by enhanced chemiluminescence (EpiZyme, Shanghai, China) and recorded with a ChemStudio gel imaging system (UVP, Upland, CA, USA). Band intensities were quantified in ImageJ and normalized to the corresponding GAPDH bands. Western blot analyses were independently repeated three times.

### 2.15. Statistical Analysis

GraphPad Prism 8.3.0 was used for data processing and figure preparation, and statistical analyses were performed in SPSS 26.0. Data are presented as mean ± standard deviation (SD). Technical replicates were averaged within each independent experiment and were not treated as independent experimental units. For animal-based analyses, the individual goat was considered the biological experimental unit. Expression across the seven matched tissues collected from the same three telogen goats was analyzed by one-way repeated-measures Analysis of variance (ANOVA) followed by Bonferroni-adjusted pairwise comparisons. Hair-cycle expression data from independent animals (*n* = 3 goats per period) were analyzed by one-way ANOVA followed by Tukey’s honestly significant difference (HSD) test.

For cell-based assays, all experiments were performed using the same donor-derived SHFSC preparation (donor *n* = 1). Independent experimental runs were used as the analytical units for within-model comparisons, whereas technical replicate wells within each run were averaged before statistical analysis. Thus, the three independent experimental runs should not be interpreted as independent donor-derived biological replicates. Two-group comparisons between treatment and the corresponding control were analyzed using two-tailed paired *t*-tests, whereas experiments involving three or more groups were analyzed by one-way ANOVA followed by Tukey’s HSD test. For cell-based qRT-PCR and Western blot analyses involving multiple molecular readouts, the corresponding comparisons within each assay and perturbation condition were treated as one family and adjusted using the Holm–Bonferroni procedure. For cell-cycle analyses, overexpression and knockdown datasets were analyzed separately. Within each dataset, treatment-versus-control comparisons for the G0/G1, S, and G2/M phases were performed using two-tailed paired *t*-tests, and the three phase-specific *p*-values were treated as one family and adjusted using the Holm–Bonferroni procedure. CCK-8 overexpression and knockdown datasets were analyzed separately using linear mixed-effects models with treatment, time, and their interaction as fixed effects and independent experiment as a random effect. Within each CCK-8 dataset, the four treatment-versus-control comparisons at 0, 24, 48, and 72 h were adjusted using the Šídák method. Statistical significance was set at *p* < 0.05; where multiple-comparison adjustment was applied, an adjusted *p* < 0.05 was considered significant.

## 3. Results

### 3.1. Cloning and Sequence Analysis of the ALDH1A1 ORF in Liaoning Cashmere Goat

**Figure 1:**
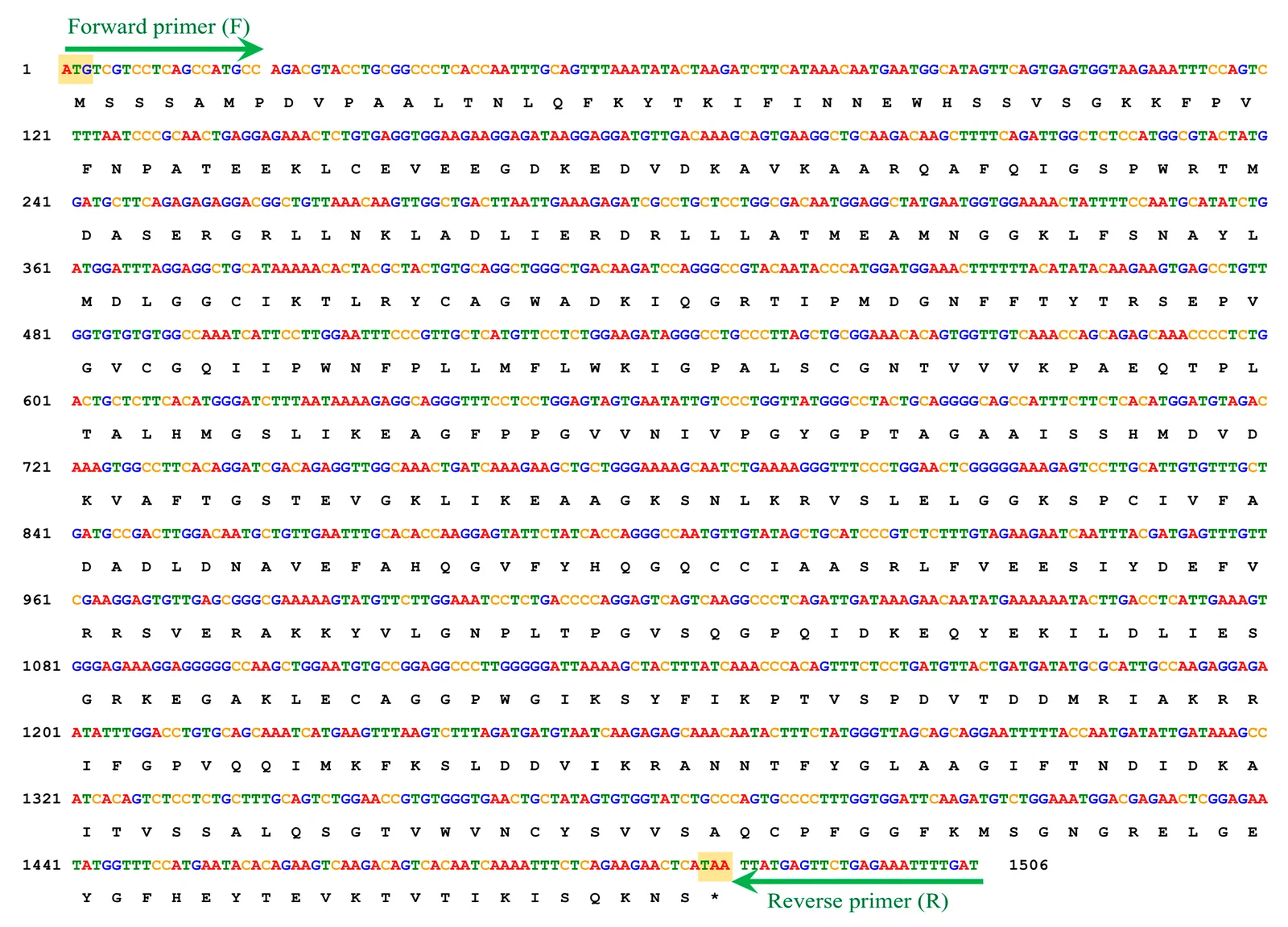
Obtained nucleotide and deduced amino acid sequences of the *ALDH1A1* ORF from Liaoning cashmere goat. The *ALDH1A1* ORF comprises 1506 bp and encodes a 501-amino-acid protein. The ATG start codon and the TAA stop codon are indicated by highlighting. The nucleotide bases are color-coded (A, red; T, green; C, orange; G, blue), while the corresponding amino acids are represented by black single-letter codes. The asterisk (*) indicates the translation termination codon. The forward and reverse primers used to clone the *ALDH1A1* ORF are marked by green arrows. The sequence was deposited in GenBank under accession number PZ619936.

Gene-specific PCR using *ALDH1A1* cloning-F and *ALDH1A1* cloning-R yielded a 1506-bp fragment from Liaoning cashmere goat skin cDNA, corresponding to the complete *ALDH1A1* ORF (Figure 1 and Supplementary Figure S1). The *ALDH1A1* ORF sequence obtained in this study has been deposited in GenBank under accession number PZ619936. The sequence extended from the ATG start codon to the TAA stop codon and was predicted to encode 501 amino acids (Figure 1). The deduced protein is composed of alanine (7.8%), arginine (3.4%), asparagine (4.2%), aspartic acid (5.0%), cysteine (2.2%), glutamine (3.6%), glutamic acid (6.4%), glycine (9.6%), histidine (1.2%), isoleucine (6.0%), leucine (7.2%), lysine (7.6%), methionine (2.6%), phenylalanine (5.2%), proline (5.0%), serine (6.6%), threonine (5.0%), tryptophan (1.4%), tyrosine (2.8%), and valine (7.4%). It has a predicted molecular weight of 54.81 kDa, an isoelectric point of 6.37, and 32 potential phosphorylation sites (Supplementary Figure S2B).

**Figure 2:**
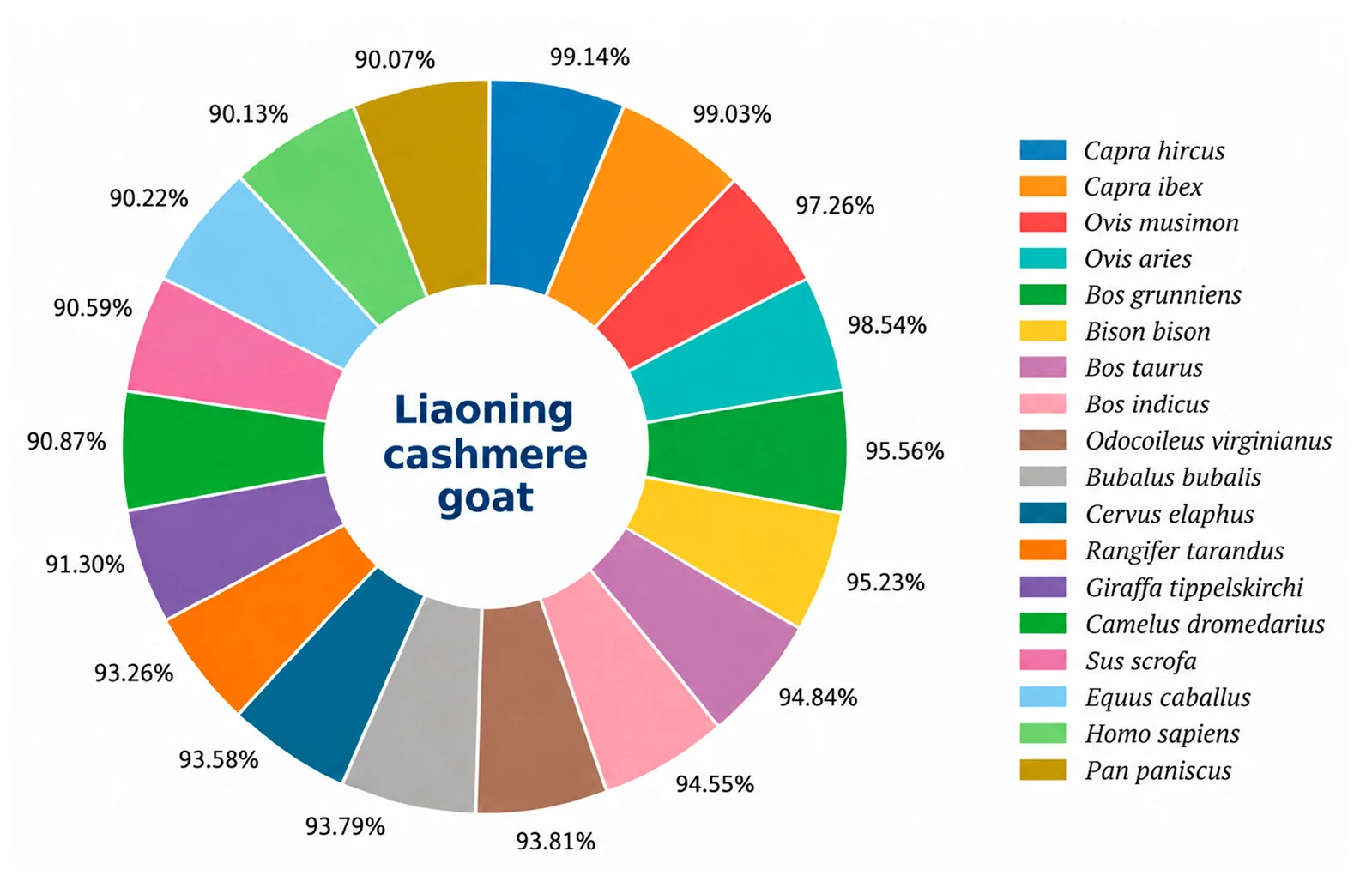
Nucleotide sequence identity of the *ALDH1A1* ORF from Liaoning cashmere goat compared with representative mammalian *ALDH1A1* sequences. The circular chart shows the percentage nucleotide identity between the experimentally obtained *ALDH1A1* ORF and the corresponding GenBank sequences: *Capra hircus* (XM_005683753.3), *Capra ibex* (JT630806.1), *Ovis aries* (NM_001009778.1), *Ovis musimon* (MT799837), *Bos grunniens* (XM_027549472.1), *Bison bison* (XM_005887845.3), *Bos taurus* (XM_061425548.1), *Bos indicus* (XM_019966069.2), *Odocoileus virginianus* (XM_020878645.2), *Bubalus bubalis* (XM_006067683.3), *Cervus elaphus* (XM_043891503.1), *Rangifer tarandus* (XM_081505450.1), *Giraffa tippelskirchi* (XM_081505448.1), *Camelus dromedarius* (XM_010999961.3), *Sus scrofa* (XM_021064911.2), *Equus caballus* (NM_001434683.1), *Homo sapiens* (NM_000689.5), and *Pan paniscus* (XM_003822194.7). Values around the chart indicate percentage nucleotide identity.

NCBI BLASTN (version 2.17.0+) analysis against GenBank showed that the cloned sequence was highly similar to the predicted goat *ALDH1A1* sequence (XM_005683753.3) (Supplementary Figure S3). Comparison with corresponding mammalian sequences showed 90.07–99.14% nucleotide identity for the *ALDH1A1* ORF (Figure 2). The highest nucleotide identity was observed with the *Capra hircus* reference sequence XM_005683753.3 (99.14%), whereas identity with the other mammalian *ALDH1A1* sequences included in the comparison ranged from 90.07% to 99.03% (Figure 2).

### 3.2. Sequence Differences and Structural Analysis of ALDH1A1 in Liaoning Cashmere Goat

**Figure 3:**
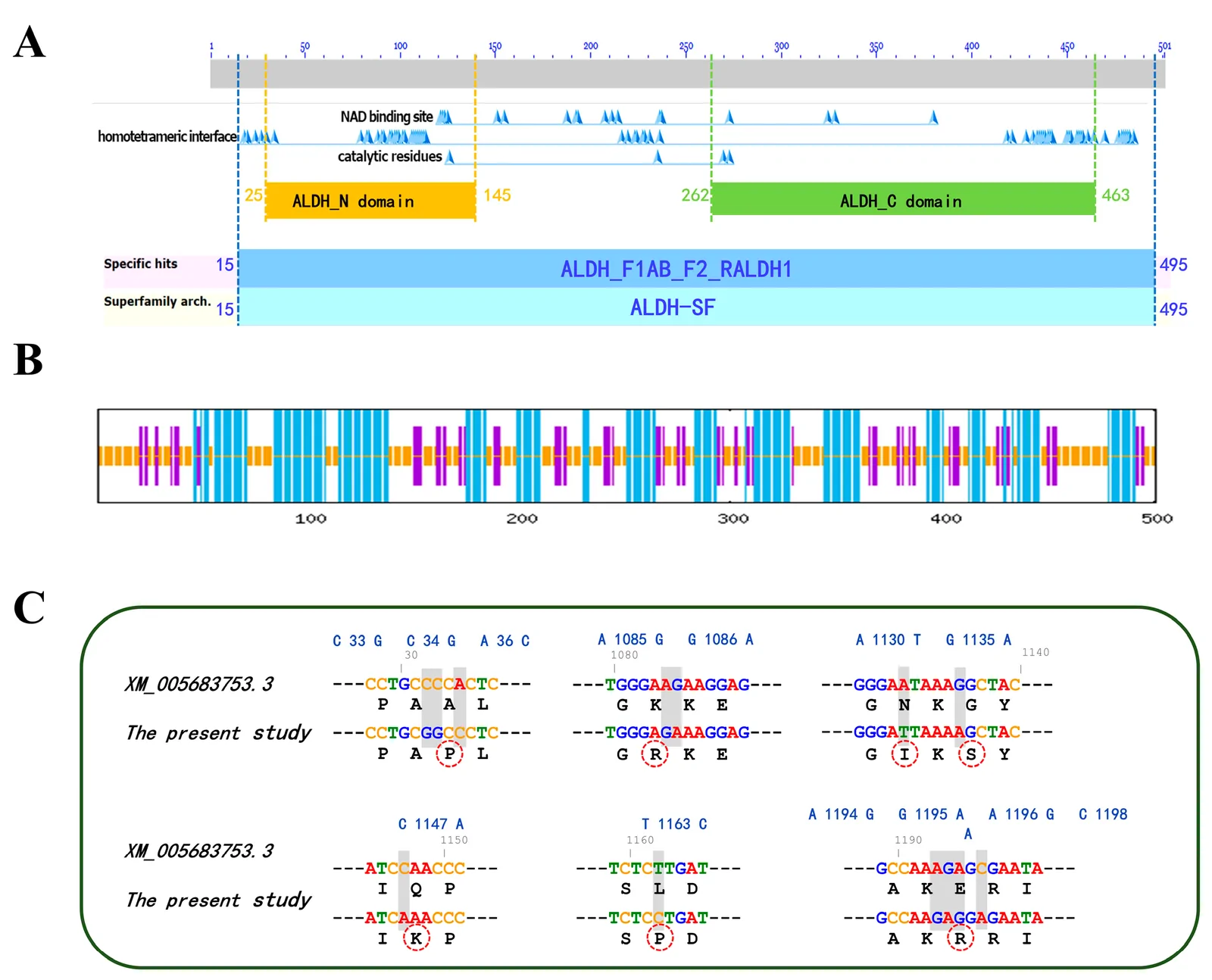
Sequence comparison and structural characterization of ALDH1A1 from Liaoning cashmere goat. (**A**) Predicted functional conserved domains of ALDH1A1 protein identified using the NCBI Conserved Domain Database. (**B**) Predicted secondary-structure composition of ALDH1A1 obtained using SOPMA. The vertical lines, arranged from longest to shortest, represent the three main types of protein secondary structure: α-helices (blue), extended strands (purple), and random coils (orange). (**C**) Alignment showing thirteen nucleotide differences between the experimentally obtained *ALDH1A1* ORF and the *Capra hircus* reference transcript XM_005683753.3, together with the corresponding differences in the deduced amino acid sequences. Gray shading highlights regions containing nucleotide sequence differences, while red dotted circles indicate the corresponding amino acid substitutions.

CDD analysis mapped residues 15–495 of Liaoning cashmere goat ALDH1A1 to the ALDH_F1AB_F2_RALDH1 region of the aldehyde dehydrogenase superfamily (ALDH-SF). This region includes the conserved ALDH_N (residues 25–145) and ALDH_C (residues 262–463) domains, together with predicted nicotinamide adenine dinucleotide (NAD)-binding sites, catalytic residues, and homotetrameric interface residues (Figure 3A), a domain organization consistent with an aldehyde dehydrogenase family protein. SOPMA estimated 202 residues in α-helical conformations (40.32%), 86 residues in β-strands (17.17%), and 213 residues in random coils (42.51%) (Figure 3B). SignalP 6.0 and TMHMM 2.0 predicted neither an N-terminal signal peptide nor transmembrane helices (Supplementary Figure S2A,C).

Comparison of the experimentally obtained 1506-bp *ALDH1A1* ORF with the *Capra hircus* reference transcript XM_005683753.3 revealed thirteen nucleotide differences: C33G, C34G, A36C, A1085G, G1086A, A1130T, G1135A, C1147A, T1163C, A1194G, G1195A, A1196G, and C1198A, based on positions in the experimentally obtained ORF sequence (Figure 3C). These differences corresponded to 99.14% nucleotide identity between the two sequences. Comparison of the deduced amino acid sequences also identified several corresponding amino acid differences between the experimentally obtained and reference sequences (Figure 3C). At the codon level, the thirteen nucleotide differences affected ten codons: three codons were synonymous (involving changes at positions 33, 1194, and 1198), whereas seven codons encoded amino acid substitutions. Notably, ten of the thirteen nucleotide differences were clustered within the 114-bp interval from positions 1085 to 1198. Because the ORF was amplified using conventional Taq polymerase, and because independent high-fidelity re-amplification and genomic DNA validation were not performed, amplification- or sequencing-derived errors cannot be completely excluded. Accordingly, these positions are reported descriptively as sequence differences relative to the reference transcript rather than as validated genomic variants.

**Figure 4:**
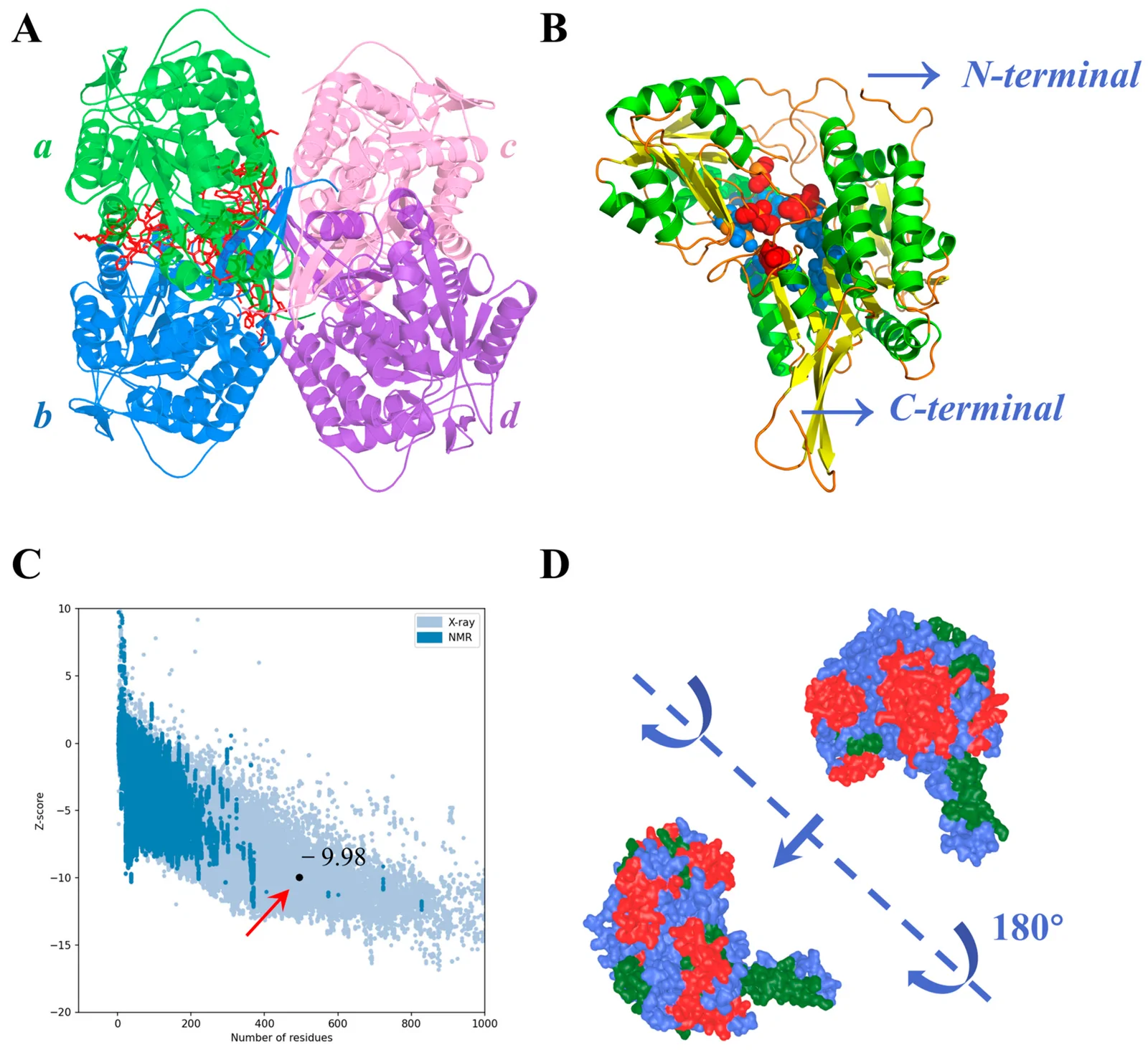
Prediction and analysis of the three-dimensional structure of ALDH1A1 protein from Liaoning cashmere goat. (**A**) The predicted homotetrameric model of ALDH1A1 protein. The four monomeric subunits are labeled a–d and displayed in green (a), blue (b), pink (c), and purple (d), respectively. The putative interface residues between subunits a and b are displayed as red stick models. (**B**) Monomeric model representing one subunit of the theoretical homotetrameric model of goat ALDH1A1 protein. The N- and C-termini represent the amino-terminal and carboxy-terminal ends of the protein, respectively, as indicated by arrows. The α-helices, β-strands, and loop regions are shown in green, yellow, and orange, respectively. The putative NAD-binding residues are displayed as blue spherical models and the putative catalytic residues (N170, E269, G300, and C303) are displayed as red spherical models. (**C**) Quality evaluation of the theoretical homotetrameric model of goat ALDH1A1 protein. The location of the z-score is highlighted as a black dot and is marked by a red arrow. (**D**) Surface representation of the predicted three-dimensional structure of ALDH1A1. The α-helices, β-strands, and random coils are presented in red, green, and blue, respectively.

Homology modeling with SWISS-MODEL automatically selected the human ALDH1A1 crystal structure (Protein Data Bank ID: 7JWW, chain D; resolution, 1.60 Å) as the optimal template. The template shared 91.42% sequence identity with the target and covered the complete 501-amino-acid sequence. The resulting model had a Global Model Quality Estimate (GMQE) of 0.93 and a QMEANDisCo Global score of 0.87 ± 0.05. The modeled goat ALDH1A1 protein showed a highly similar overall fold to the template and was predicted to form a homotetramer composed of four monomeric subunits, with putative a–b interaction surfaces between subunits (Figure 4A). Each modeled subunit contained the conserved ALDH domain and corresponding predicted functional sites. One representative monomer is shown in Figure 4B, including the predicted NAD-binding sites and catalytic residues N170, E269, G300, and C303.

Independent model-quality assessments further supported the structural model. ProSA-web yielded a z-score of −9.98, within the range observed for experimentally resolved proteins of comparable size (Figure 4C). MolProbity analysis yielded a score of 0.89 and a clashscore of 0.66; 96.95% of residues were located in favored Ramachandran regions, with 0.20% classified as Ramachandran outliers and 0.49% as rotamer outliers. No bad bonds were detected. The predicted ALDH1A1 structure is also presented as a surface model in Figure 4D, with α-helices, β-strands, and random coils shown in red, green, and blue, respectively; the two views are separated by a 180° rotation.

### 3.3. Phylogenetic Analysis of ALDH1A1 Protein from Liaoning Cashmere Goat

**Figure 5:**
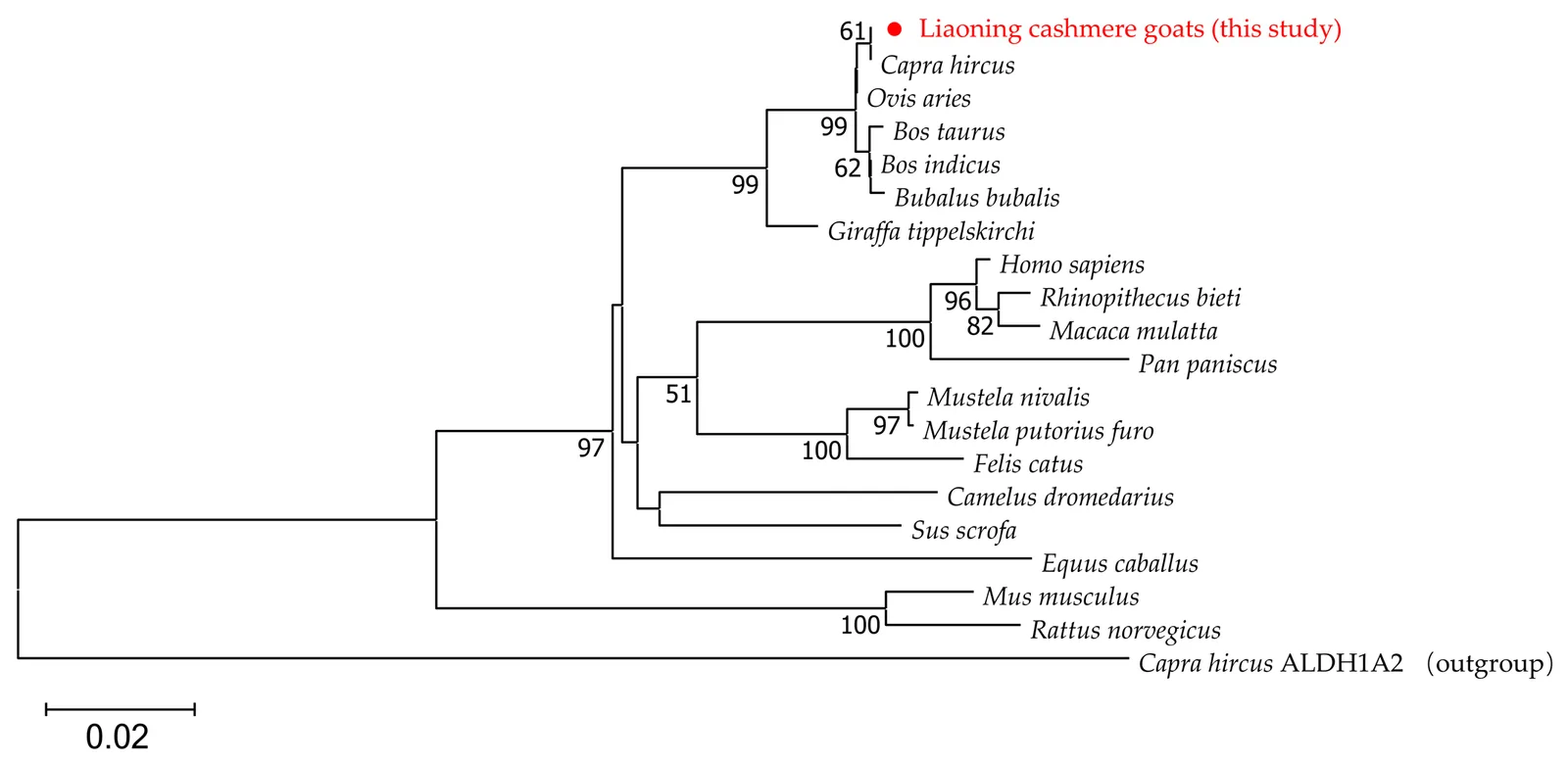
Phylogenetic relationships of ALDH1A1 proteins across mammalian species. The neighbor-joining tree was constructed based on ALDH1A1 amino acid sequences using the p-distance method with 1000 bootstrap replicates. Bootstrap values are shown at the branch nodes, and branch lengths represent evolutionary distances. The Liaoning cashmere goat ALDH1A1 sequence obtained in this study is highlighted in red. The GenBank accession numbers of the sequences used for phylogenetic analysis are indicated as follows: Liaoning cashmere goat (this study), *Capra hircus* (XP_005683810.2), *Ovis aries* (NP_001009778.1), *Bos taurus* (NP_776664.1), *Bos indicus* (XP_019821628.1), *Bubalus bubalis* (XP_006067745.1), *Giraffa tippelskirchi* (XP_081361576.1), *Homo sapiens* (NP_000680.2), *Rhinopithecus bieti* (XP_017711271.1), *Macaca mulatta* (XP_017782232.1), *Pan paniscus* (XP_034823450.1), *Mustela nivalis* (XP_082552722.1), *Mustela putorius furo* (XP_004769259.1), *Felis catus* (XP_003995456.1), *Camelus dromedarius* (XP_010998263.1), *Sus scrofa* (XP_020920570.1), *Equus caballus* (NP_001421612.1), *Mus musculus* (NP_001348342.1), *Rattus norvegicus* (XP_063136020.1), and *Capra hircus* ALDH1A2 (XP_017909759.1, outgroup).

Phylogenetic analysis of representative ALDH1A1 amino acid sequences revealed the clustering pattern of mammalian ALDH1A1 proteins (Figure 5). The Liaoning cashmere goat ALDH1A1 sequence clustered closely with the *Capra hircus* reference sequence and was positioned within a mammalian ruminant-associated group together with *Ovis aries*, *Bos taurus*, and other ruminant species. Because several internal nodes showed relatively low bootstrap support, no further fine-scale evolutionary relationships were inferred. The GenBank accession numbers of the sequences included in the phylogenetic analysis are provided in the Figure 5 legend.

### 3.4. Expression Patterns of the ALDH1A1 mRNA and Protein in Liaoning Cashmere Goats

**Figure 6:**
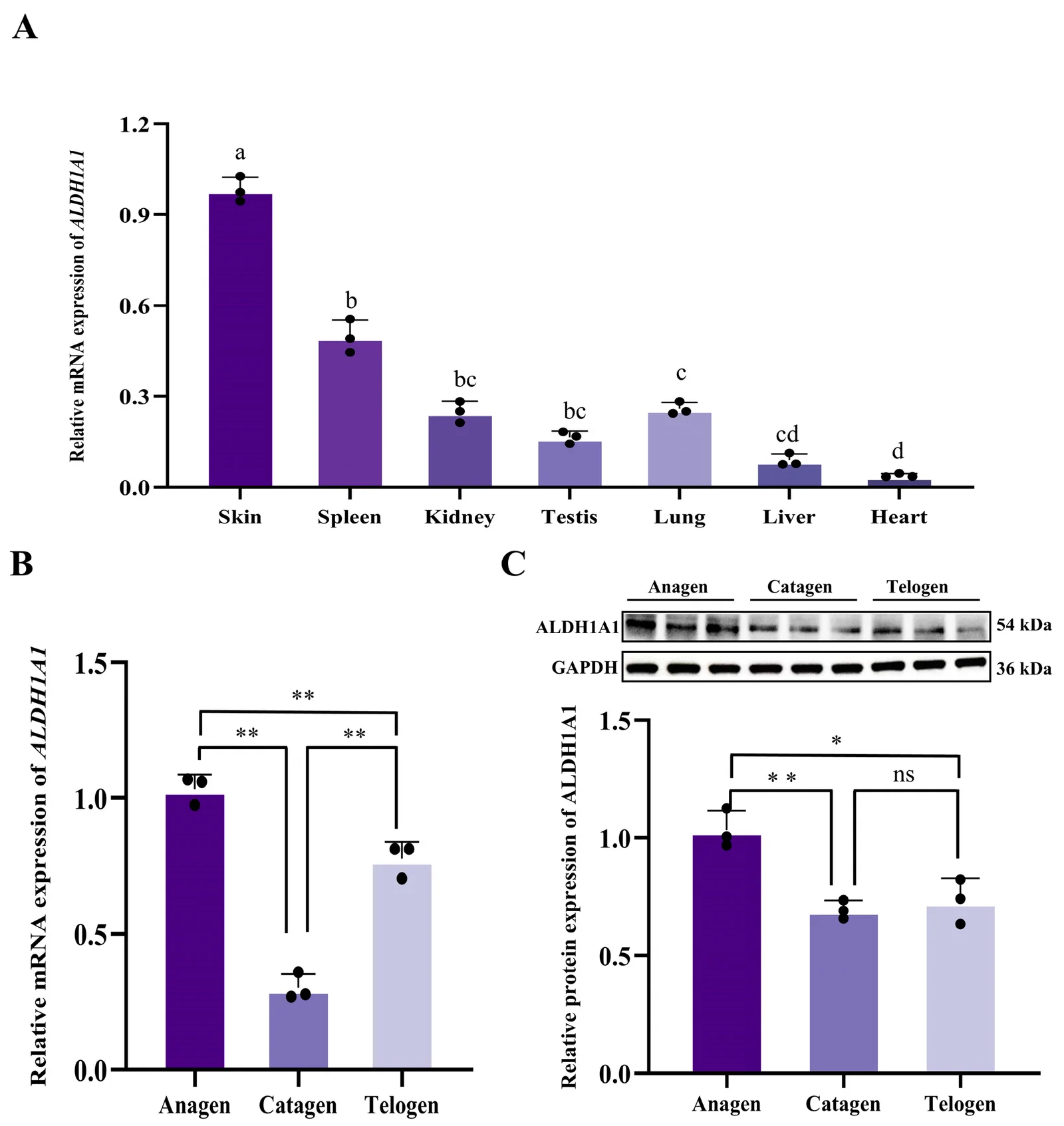
Expression patterns of *ALDH1A1* mRNA and protein from Liaoning cashmere goats. (**A**) Relative expression of *ALDH1A1* mRNA across seven tissues collected from the same three telogen goats. Different lowercase letters indicate significant differences (*p* < 0.05). (**B**) *ALDH1A1* mRNA expression in skin samples from the seasonally assigned anagen, catagen, and telogen periods. (**C**) Representative Western blots and relative quantification of ALDH1A1 protein across the seasonally assigned anagen, catagen, and telogen periods. GAPDH served as the loading control. * *p* < 0.05; ** *p* < 0.01; ns, not significant. Data are presented as mean ± SD. Individual points represent individual goats. For panel (**A**), each of the same three telogen goats contributed one sample of each of the seven tissues (*n* = 3 goats); for panels (**B**,**C**), three independent goats were analyzed per period (*n* = 3 per period; nine goats in total). Statistical analysis: repeated-measures ANOVA with Bonferroni correction for (**A**), and one-way ANOVA with Tukey’s HSD test for (**B**,**C**). qRT-PCR measurements were performed in technical triplicate. Uncropped blots are shown in Supplementary Figure S4.

Across the seven tissues collected from the three telogen goats, qRT-PCR showed that *ALDH1A1* transcript abundance was highest in skin (*p* < 0.05) (Figure 6A). Skin samples collected during the seasonally assigned anagen, catagen, and telogen periods were analyzed by qRT-PCR and Western blotting (Figure 6B,C). *ALDH1A1* mRNA abundance was highest during the seasonally assigned anagen period, followed by telogen and catagen; all pairwise comparisons were significant (*p* < 0.01) (Figure 6B). The protein profile was generally consistent with the transcript data. ALDH1A1 protein abundance was higher in anagen than in catagen (*p* < 0.01) and telogen (*p* < 0.05), whereas catagen and telogen did not differ significantly (*p* > 0.05) (Figure 6C).

### 3.5. Morphological and Immunofluorescence Characterization of SHFSCs

**Figure 7:**
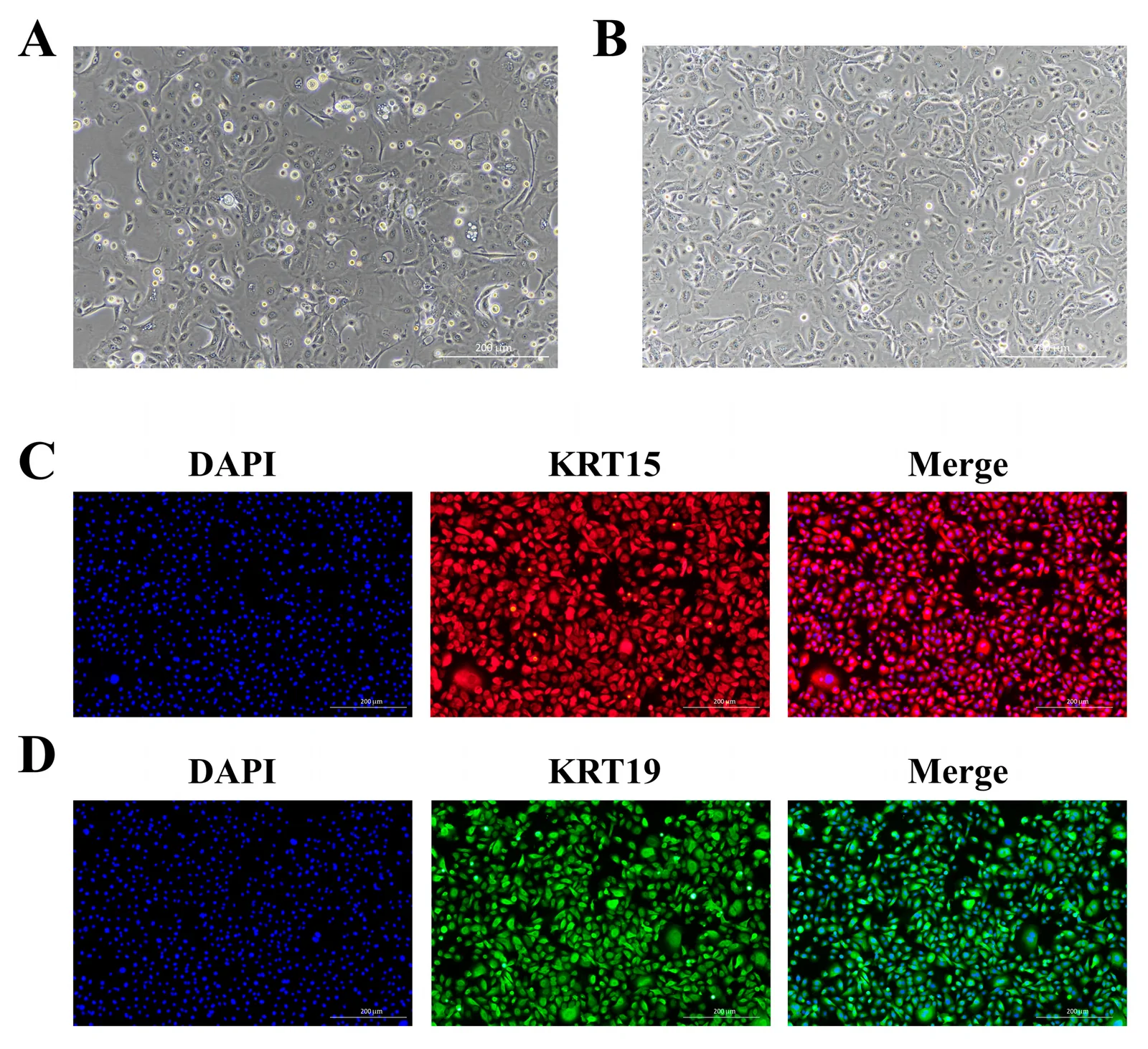
Morphological and immunofluorescence characterization of primary SHFSCs. (**A**) Representative bright-field image of cells during early primary culture. (**B**) Representative bright-field image of cells after passage. (**C**) Immunofluorescence staining of KRT15 in P1 cells. Blue, DAPI-stained nuclei; red, KRT15-positive staining; Merge, merged fluorescence channels. (**D**) Immunofluorescence staining of KRT19 in P1 cells. Blue, DAPI-stained nuclei; green, KRT19-positive staining; Merge, merged fluorescence channels. Images were acquired at an original magnification of 100×; scale bars are shown in the corresponding panels. Corresponding negative controls showed no specific staining. The cells were derived from the single donor goat used in this study (donor *n* = 1).

Representative bright-field images showed adherent growth of the cells during early primary culture and after passage (Figure 7A,B). Immunofluorescence staining of P1 cells showed clear positive signals for KRT15 and KRT19 (Figure 7C,D), whereas the corresponding negative controls showed no specific staining. Together with their origin from isolated secondary hair follicles and characteristic adherent morphology, these findings provided phenotypic support consistent with the SHFSC identity of the cultured cell preparation used in this study.

### 3.6. Role of ALDH1A1 in SHFSC Proliferation

**Figure 8:**
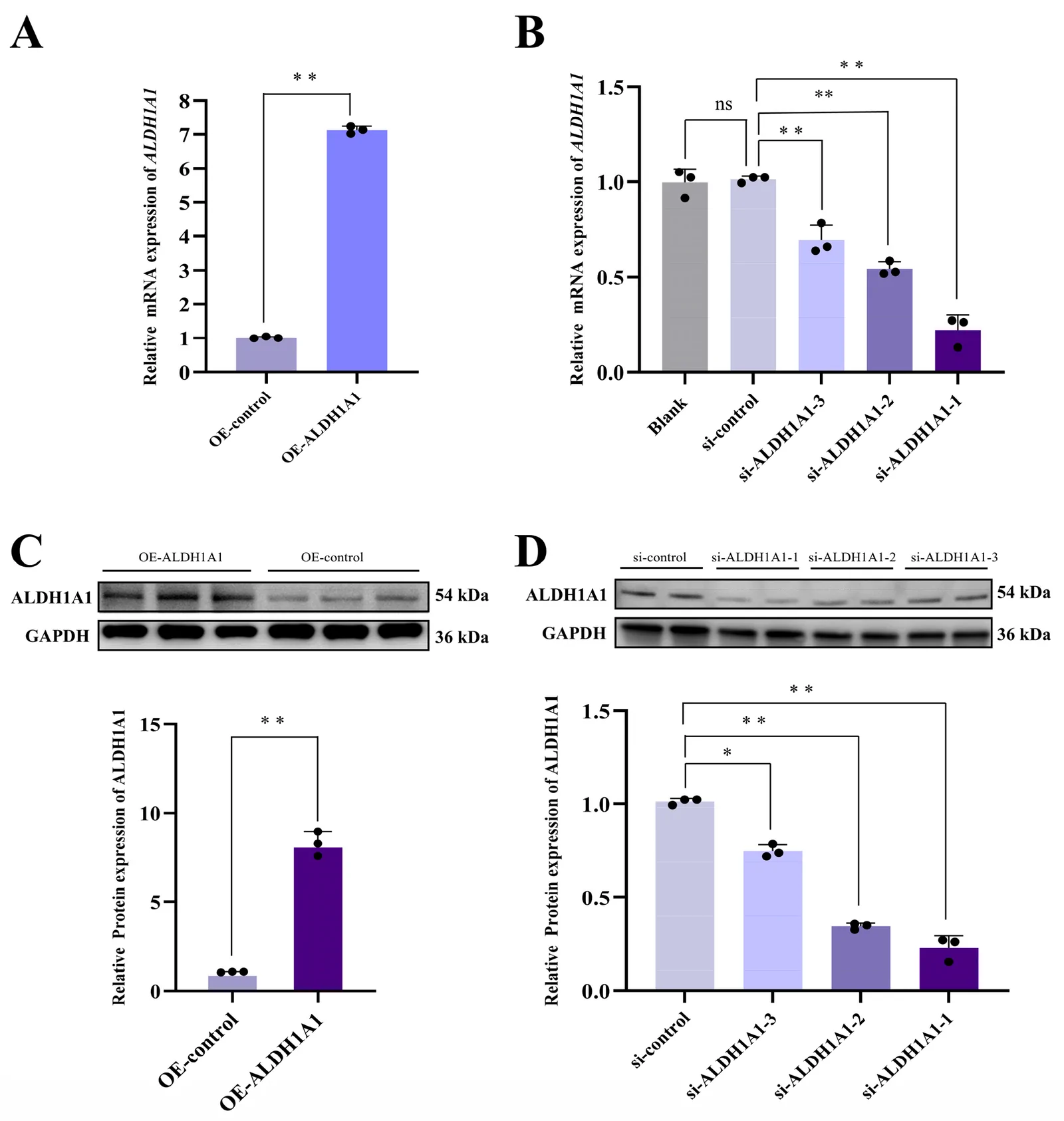
Optimization and validation of *ALDH1A1* overexpression and knockdown conditions in SHFSCs. (**A**) Validation of *ALDH1A1* overexpression at the mRNA level. (**B**) Efficiency of *ALDH1A1* siRNA transfection at the mRNA level. (**C**) Validation of *ALDH1A1* overexpression at the protein level. (**D**) Efficiency of *ALDH1A1* siRNA transfection at the protein level. Statistical analysis: two-tailed paired *t*-tests were used for the two-group overexpression comparisons (**A**,**C**), whereas one-way ANOVA followed by Tukey’s HSD test was used for the multi-group knockdown comparisons (**B**,**D**). Blank, reagent-only control. Data are shown as mean ± SD from three independent experimental repeats using primary SHFSCs derived from a single donor goat (donor *n* = 1). qRT-PCR measurements were performed in technical triplicate. * *p* < 0.05; ** *p* < 0.01; ns, not significant. The Western blot images in panels (**C**,**D**) are representative of one experimental run, whereas the quantitative points summarize three independent experimental repeats. Representative uncropped blots from one experimental run are shown in Supplementary Figure S7.

Preliminary optimization showed that 0.5 μg/well pcDNA3.1(+)-*ALDH1A1* produced a reproducible increase in *ALDH1A1* expression while maintaining normal cell morphology and attachment. *ALDH1A1* mRNA and protein abundance increased by approximately seven- to eightfold relative to the OE-control. No overt morphological deterioration or obvious increase in detached or floating cells was observed under the evaluated conditions. Therefore, 0.5 μg/well was selected for subsequent overexpression experiments (Figure 8A,C). Preliminary concentration optimization identified 100 nM as a condition that produced stable *ALDH1A1* suppression without overt deterioration in cell morphology or attachment. Under this condition, the three *ALDH1A1*-targeting siRNAs were compared directly. si-*ALDH1A1*-1 produced the strongest and most reproducible reduction in *ALDH1A1* expression at both the mRNA and protein levels and was therefore selected as the primary knockdown reagent for subsequent experiments (Figure 8B,D). ALDH1A1 protein abundance was reduced by approximately 70% relative to the si-control. si-*ALDH1A1*-1 is hereafter referred to as si-*ALDH1A1*.

**Figure 9:**
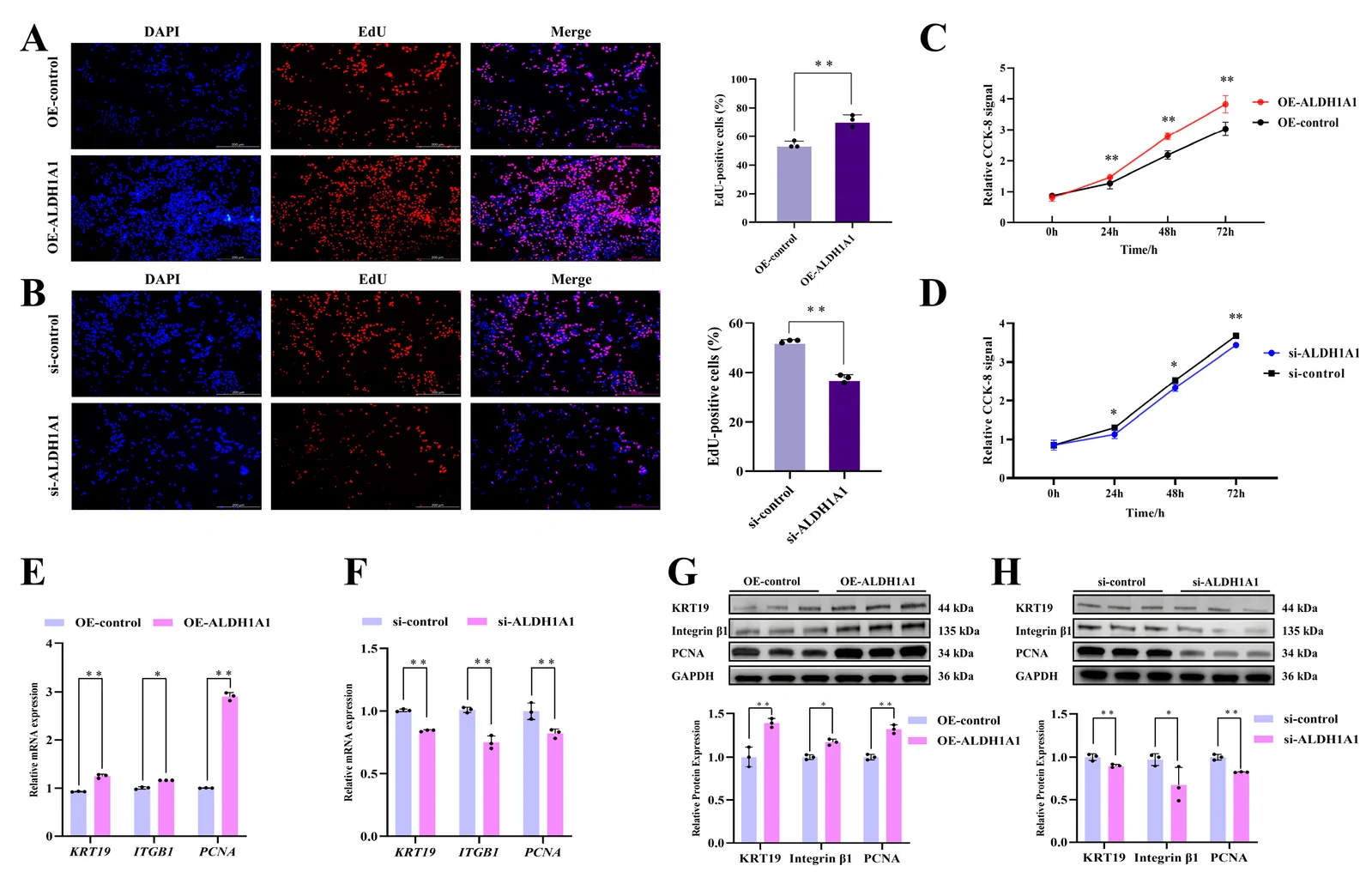
Cellular responses to *ALDH1A1* overexpression and knockdown in SHFSCs. (**A**,**B**) Representative EdU staining images and quantitative analysis of the EdU-positive cell fraction in the *ALDH1A1* overexpression group (**A**) and knockdown group (**B**). Blue, DAPI-stained nuclei; red, EdU-positive cells; Merge, merged channels. Scale bar = 200 μm. (**C**,**D**) Time-dependent CCK-8 signals in the *ALDH1A1* overexpression group (**C**) and knockdown group (**D**), reflecting changes in cellular metabolic activity. (**E**,**F**) qRT-PCR analysis of *KRT19*, *ITGB1*, and *PCNA* mRNA expression in the *ALDH1A1* overexpression group (**E**) and knockdown group (**F**). (**G**,**H**) Western blotting and densitometric analysis of KRT19, integrin β1, and PCNA protein expression in the *ALDH1A1* overexpression group (**G**) and knockdown group (**H**), with GAPDH as the internal reference protein. Statistical analysis: paired *t*-test for EdU; linear mixed-effects model with Šídák-adjusted comparisons for CCK-8; and paired *t*-tests with Holm–Bonferroni correction for qRT-PCR and Western blotting. Data are shown as mean ± SD from three independent experimental repeats using primary SHFSCs derived from a single donor goat (donor *n* = 1). Technical replicates: CCK-8, five wells per treatment/time point; EdU, three wells per treatment; qRT-PCR, three reactions per sample. Uncropped blots are shown in Supplementary Figure S8. * *p* < 0.05; ** *p* < 0.01. Adjusted *p*-values were used where multiple-comparison correction was applied.

EdU incorporation and CCK-8 measurements were used to assess DNA synthesis and cellular metabolic responses, respectively. Overexpression increased the EdU-positive fraction and CCK-8 signal (Figure 9A,C), whereas knockdown reduced both measures (Figure 9B,D). Among the *ALDH1A1*-targeting siRNAs evaluated above, si-*ALDH1A1*-2 was further used as an independent sequence to assess the reproducibility of proliferation-related responses. In the independent CCK-8 validation, si-*ALDH1A1*-2-treated cells showed lower mean absorbance values than si-control cells at 24, 48, and 72 h. However, only the reduction at 72 h reached statistical significance after Šídák adjustment (adjusted *p* = 0.00037; Supplementary Figure S6A). The corresponding CCK-8 data for both siRNAs are provided in Supplementary Data S3. In addition, si-*ALDH1A1*-2 significantly reduced the EdU-positive fraction (Supplementary Figure S6B,C). These findings provide additional support for the proliferation-related phenotype observed following *ALDH1A1* knockdown. qRT-PCR showed that *ALDH1A1* overexpression increased *KRT19*, *ITGB1*, and *PCNA* transcript levels (Figure 9E), whereas knockdown decreased their transcript levels (Figure 9F). Western blotting showed corresponding increases in KRT19, integrin β1, and PCNA protein abundance after *ALDH1A1* overexpression (Figure 9G) and decreases after knockdown (Figure 9H). Collectively, these results indicate that increased *ALDH1A1* expression was accompanied by enhanced proliferation-related phenotypes and concordant changes in these molecular readouts, whereas *ALDH1A1* knockdown produced the opposite pattern.

### 3.7. Impact of ALDH1A1 on the Cell Cycle and Colony Formation of SHFSCs

**Figure 10:**
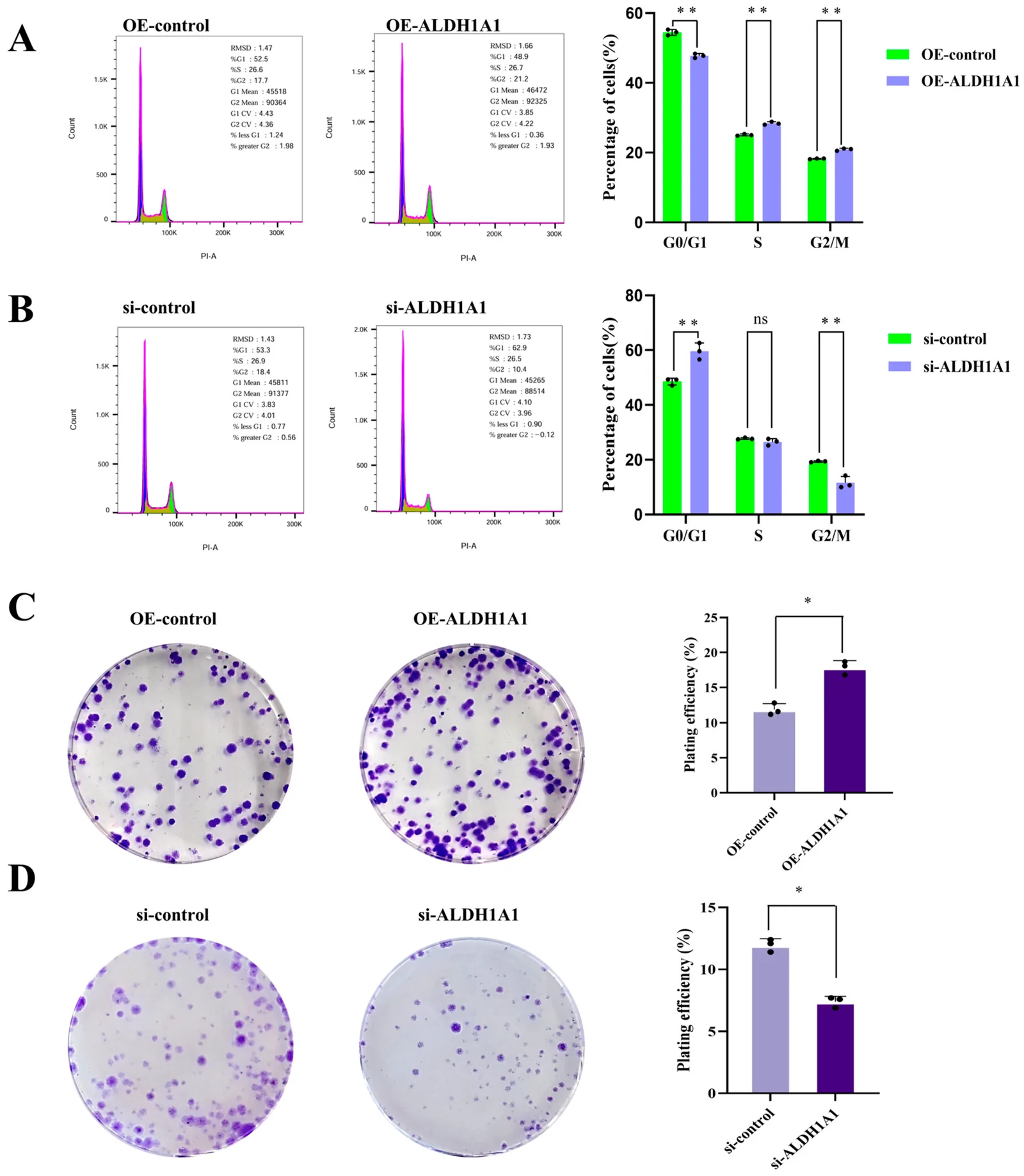
Association of *ALDH1A1* perturbation with cell-cycle distribution and clonogenic capacity of SHFSCs. (**A**) Representative flow cytometry histograms (**left**) and quantitative analysis of cell proportions in G0/G1, S, and G2/M phases after *ALDH1A1* overexpression. (**B**) Representative flow cytometry histograms (**left**) and quantitative analysis of cell proportions in G0/G1, S, and G2/M phases after *ALDH1A1* knockdown. (**C**) Representative images and quantification of plating efficiency in OE-control and OE-*ALDH1A1* groups. (**D**) Representative images and quantification of plating efficiency in si-control and si-*ALDH1A1* groups. In panels (**A**,**B**), green bars represent the control groups, and light purple bars represent the corresponding ALDH1A1-perturbed groups. In panels (**C**,**D**), light purple and dark purple bars represent the control and ALDH1A1-perturbed groups, respectively. Statistical analysis: paired *t*-tests with Holm–Bonferroni correction for cell-cycle data (**A**,**B**) and paired *t*-tests for colony-formation data (**C**,**D**). Data are shown as mean ± SD from three independent experimental repeats using primary SHFSCs derived from a single donor goat (donor *n* = 1); colony-formation assays included three technical replicate wells per treatment in each independent experimental repeat. For panels (**A**,**B**), * adjusted *p* < 0.05 and ** adjusted *p* < 0.01; for panels (**C**,**D**),* *p* < 0.05; ns, not significant.

Flow cytometry and colony-formation assays were used to assess how *ALDH1A1* overexpression and knockdown affected SHFSC cell-cycle distribution and clonogenic capacity (Figure 10). The representative gating strategy used for cell-cycle analysis, including debris exclusion and singlet discrimination, is provided in Supplementary Figure S9. Overexpression shifted the cell population away from G0/G1: the G0/G1 fraction decreased by 7.6 percentage points, while S and G2/M increased by 3.1 and 4.5 percentage points, respectively (Figure 10A). Knockdown produced the opposite shift, increasing G0/G1 by 9.6 percentage points and decreasing S and G2/M by 1.6 and 8.0 percentage points, respectively (Figure 10B). These distributions indicate that *ALDH1A1* overexpression facilitates progression beyond G0/G1, whereas knockdown favors G0/G1 accumulation and limits subsequent cell-cycle progression. Colony formation showed a parallel functional response. *ALDH1A1* knockdown reduced plating efficiency (*p* = 0.012), whereas overexpression increased plating efficiency (*p* = 0.029) (Figure 10C,D). Representative images also showed smaller, more sparsely distributed colonies after *ALDH1A1* knockdown and more densely packed colonies with clear boundaries after overexpression. Together, these findings show that experimental *ALDH1A1* perturbation was associated with changes in cell-cycle distribution and clonogenic behavior in this donor-derived SHFSC model.

## 4. Discussion

Annual cycling of secondary hair follicles supports the continuous production of cashmere fibers in cashmere goats. During anagen, SHFSC activation and proliferation accompany active follicular growth, whereas these cells are comparatively quiescent during catagen and telogen [3,5,32]. This cyclical process is influenced by multiple endogenous signals [33,34], making the identification of relevant molecular regulators important for understanding cashmere fiber growth. ALDH1A1 is a retinaldehyde dehydrogenase involved in a key rate-limiting step of retinoic acid (RA) biosynthesis and has been linked to HFSC activation [28,35,36]. However, the sequence features, expression pattern, and cellular effects of *ALDH1A1* in cashmere goat SHFSCs remain poorly defined. We therefore cloned the complete *ALDH1A1* ORF from Liaoning cashmere goats, characterized its tissue-expression pattern, and tested its effects on SHFSC proliferation in vitro.

The cloned *ALDH1A1* ORF was 1506 bp long and was predicted to encode 501 amino acids. Across the mammalian sequences examined, the Liaoning cashmere goat *ALDH1A1* ORF showed 90.07–99.14% nucleotide identity. Its predicted molecular weight (54.81 kDa) and isoelectric point (6.37) fall within values reported for orthologous ALDH1A1 proteins from other mammals, including yak and human [37,38], supporting the identity of the cloned sequence. The high cross-species conservation is also compatible with the conserved biological functions reported for ALDH1A1 [39,40,41,42]. Because these differences were identified from a cDNA-derived ORF amplified using Premix Taq, and neither independent high-fidelity re-amplification nor genomic DNA validation was performed, they are interpreted only as reference-relative sequence differences; amplification- or sequencing-derived artifacts cannot be completely excluded. Structural analysis placed residues 15–495 in the canonical ALDH_F1AB_F2_RALDH1 region of the aldehyde dehydrogenase superfamily (ALDH-SF). This region contains the ALDH_N (residues 25–145) and ALDH_C (residues 262–463) subdomains, together with predicted NAD-binding sites, catalytic residues, and homotetrameric interface residues, which is consistent with classification of the deduced protein as an aldehyde dehydrogenase family member [43]. The predicted secondary structure contains α-helices, β-sheets, and random coils. SignalP 6.0 and TMHMM 2.0 detected no N-terminal signal peptide or transmembrane helices, respectively, consistent with the known intracellular localization of ALDH1A1 [44]. NetPhos 3.1 identified 32 potential phosphorylation sites. ALDH1A1 and ALDH1A3 have also been associated with skeletal muscle satellite-cell differentiation [45], suggesting that ALDH1-family proteins may have broader roles in adult stem and progenitor cell systems.

Among the tissues analyzed, *ALDH1A1* transcripts were most abundant in skin, consistent with previous evidence of dynamic retinoid metabolism in skin and hair follicles [28]. In samples assigned according to the established seasonal hair-cycle pattern of Liaoning cashmere goats, *ALDH1A1* mRNA abundance was highest during anagen, and the protein-expression pattern was broadly consistent with the transcript data. HFSCs undergo substantial changes in proliferative and self-renewal states during the hair cycle [46,47]. Recent single-cell analyses in goats have further revealed hair-cycle-associated cellular heterogeneity and apoptosis-related programs [48]. In addition, the hair-follicle mesenchymal niche contributes to the maintenance of cellular quiescence and survival [49]. Previous immunolocalization studies in mouse hair follicles detected ALDH1A1 in the bulge region during anagen [28], while previous CRISPR/Cas9-based screening and functional analysis in cashmere goat HFSCs demonstrated that *ALDH1A1* is required for normal HFSC proliferation [29]. Together, these observations provided the biological rationale for examining *ALDH1A1* function in cultured SHFSCs. However, the tissue-expression analyses in the present study were performed on whole skin and did not include histological staging or in situ localization of ALDH1A1. Accordingly, the observed differences in whole-skin *ALDH1A1* abundance cannot be assigned specifically to SHFSCs and may also reflect differences in follicular density or cellular composition among sampling periods. In addition, because the three sampling periods occurred in different seasons, hair-cycle stage coincided with changes in environmental factors such as photoperiod and temperature. The present design therefore cannot fully distinguish hair-cycle-associated variation in *ALDH1A1* expression from seasonal environmental influences. The subsequent SHFSC experiments were used to evaluate the cellular response to altered *ALDH1A1* expression in vitro and should not be interpreted as identifying SHFSCs as the in situ source of ALDH1A1 in skin. Future studies incorporating histological staging, follicular morphometry, and in situ co-localization of ALDH1A1 with validated SHFSC markers will be required to address these questions directly.

In SHFSCs, at the transcript level, *ALDH1A1* overexpression increased *KRT19*, *ITGB1*, and *PCNA* expression, whereas knockdown reduced their expression. Western blotting showed corresponding changes in KRT19, integrin β1, and PCNA protein abundance. KRT19 is a keratin component of the epithelial cytoskeleton, whereas integrin β1 participates in cell–matrix adhesion and related cellular signaling. PCNA is closely associated with DNA replication and proliferative activity, consistent with the EdU and cell-cycle findings [14]. The CCK-8 and EdU results showed concordant responses: overexpression increased the CCK-8 signal and EdU incorporation, whereas knockdown decreased both. Because CCK-8 primarily reflects cellular reductive/metabolic activity, this readout was interpreted together with the EdU and other proliferation-related findings. Direct cell counting, apoptosis assays, and differentiation-marker analyses were not performed; therefore, contributions from altered cell survival or differentiation cannot be excluded from the observed phenotype. In other cellular systems, *ALDH1A1* has been linked to granulosa-cell proliferation and to AKT activation and stem-like properties in esophageal squamous cell carcinoma [50,51]. SHFSC clonogenicity changed in the same direction, with plating efficiency increasing after *ALDH1A1* overexpression and decreasing after knockdown. Because colony formation reflects the sustained proliferative and clonogenic capacity of individual epithelial cells in vitro [52,53], this result is also consistent with reports linking *ALDH1A1* to colony formation in melanoma cells [54]. Cell-cycle analysis further showed that overexpression reduced the G0/G1 fraction and increased both S and G2/M, while knockdown increased G0/G1 and reduced G2/M. HFSC metabolic state is closely related to cell-cycle transitions and hair follicle regeneration [33,55,56], and stress-induced G0/G1 accumulation has been associated with impaired HFSC activation [57,58]. Taken together, the metabolic activity, DNA synthesis, colony formation, and cell cycle findings consistently support a positive role for *ALDH1A1* in proliferation-related phenotypes of cashmere goat SHFSCs.

The perturbation conditions used in this study were selected following preliminary optimization, with morphological tolerability considered alongside the reproducibility of *ALDH1A1* modulation. The selected overexpression and knockdown conditions produced consistent changes in *ALDH1A1* expression without overt deterioration in cell morphology or attachment. The independent siRNA si-*ALDH1A1*-2 also reduced the CCK-8 signal at 72 h and EdU incorporation, providing partial support for the reproducibility of the knockdown-associated proliferation-related phenotype. Nevertheless, several limitations should be considered. si-*ALDH1A1*-2 was not evaluated across the complete set of molecular-response, colony-formation, and cell-cycle assays, and an siRNA-resistant rescue experiment was not included. Therefore, additional validation using rescue strategies or complementary approaches would further strengthen the causal interpretation of *ALDH1A1* function. Although *ALDH1A1* suppression was confirmed at 48 h after transfection, *ALDH1A1* expression was also reassessed in separate parallel cultures maintained to a time point corresponding to the end of the colony-formation assay. At this later time point, the reduction relative to si-control was weak and no longer statistically significant. Because these measurements were obtained from parallel cultures rather than the wells used for colony quantification, they do not establish sustained *ALDH1A1* suppression throughout the assay. Accordingly, the colony-formation result should be interpreted as a phenotype observed following the initial siRNA treatment rather than as evidence of persistent knockdown throughout the entire 5–7-day assay period. In addition, related ALDH family members, including *ALDH1A2* and *ALDH1A3*, were not directly evaluated in the present study; therefore, the results obtained with a second independent siRNA do not establish *ALDH1A1*-specific causality. ALDH1A1 protein abundance after plasmid overexpression was not directly calibrated against endogenous ALDH1A1 abundance in anagen skin, and the overexpression condition should therefore be interpreted as an experimental gain-of-function model rather than as a physiological mimic of anagen-level *ALDH1A1* expression. Phenotypic characterization was limited to KRT15 and KRT19 immunofluorescence, and a broader goat-validated marker panel was not assessed; therefore, SHFSC identity should be regarded as phenotypically supported rather than definitively established. Finally, all cellular functional assays were performed using primary SHFSCs derived from a single donor goat (*n* = 1), precluding assessment of inter-individual variability. Thus, the cellular findings should be interpreted within the context of this donor-derived SHFSC model, and validation using independent SHFSC preparations from additional animals will be required to assess their generalizability.

ALDH1A1 generates RA by oxidizing retinaldehyde. Previous studies indicate that RA can promote the transition of HFSCs from quiescence to activation through Wnt/β-catenin signaling [26], while both Wnt/β-catenin and Phosphoinositide 3-kinase/Protein kinase B (PI3K/AKT) pathways contribute to HFSC activation and hair follicle regeneration [17,19,59,60,61]. Associations between ALDH1A1, AKT activation, and β-catenin-related processes have also been reported in non-follicular models such as esophageal squamous cell carcinoma and melanoma [51,54]. Together, these previous findings provide a biologically plausible basis for hypothesizing that RA-related signaling may contribute to the proliferative phenotype associated with *ALDH1A1* in SHFSCs. However, the present study did not measure ALDH enzymatic activity, intracellular RA concentrations, Retinoic acid receptor (RAR) signaling, active β-catenin, or PI3K/AKT activity, and did not include rescue experiments or experiments using a catalytically inactive ALDH1A1 mutant. Therefore, the present data do not establish that the *ALDH1A1*-associated proliferative phenotype is mediated by RA biosynthesis or downstream RA-related signaling. Future studies combining measurements of ALDH activity and intracellular RA with rescue experiments using exogenous RA or an siRNA-resistant *ALDH1A1* construct, comparison with a catalytically inactive ALDH1A1 mutant, and targeted analyses of RAR signaling, Wnt/β-catenin signaling, and PI3K/AKT signaling will be required to test this hypothesis and distinguish enzymatic from non-enzymatic effects of ALDH1A1.

## 5. Conclusions

In this study, the complete *ALDH1A1* coding sequence was obtained from Liaoning cashmere goats and its major molecular and phylogenetic features were characterized. *ALDH1A1* expression was enriched in skin and reached its highest level during the seasonally assigned anagen period. In SHFSCs, *ALDH1A1* overexpression increased cellular metabolic activity, DNA synthesis, clonogenic capacity, and cell-cycle progression, whereas knockdown produced the opposite pattern. These results support an association between experimental *ALDH1A1* perturbation and proliferation-related phenotypes within the single-donor primary SHFSC model used in this study, but they do not establish *ALDH1A1*-specific causality or generalizability across animals. Whether these phenotypes are mediated by ALDH enzymatic activity, RA biosynthesis, or downstream RA-related signaling remains to be determined.

## Data Availability

The data supporting the findings of this study are available within the article and its Supplementary Materials. The *ALDH1A1* ORF sequence generated in this study has been deposited in GenBank under accession number PZ619936. Additional supporting data, including representative uncropped Western blot images, source microscopy images, colony-formation source images, representative flow cytometry FCS files from one independent experimental run, and blank-corrected CCK-8 data for both *ALDH1A1*-targeting siRNAs, are provided in the Supplementary Materials.

## Supplementary Materials

The following supporting information can be downloaded at: https://www.mdpi.com/article/10.3390/cells15201865/s1. Table S1: Primer sequences used in this study; Table S2: Software, databases, and online resources used for sequence and structural analyses in this study; Table S3: siRNA sequences used for *ALDH1A1* knockdown; Table S4: Primary and secondary antibodies used in this study; Figure S1: PCR amplification of the full-length *ALDH1A1* coding sequence from Liaoning cashmere goat skin cDNA; Figure S2: In silico prediction of the Liaoning cashmere goat ALDH1A1 protein; Figure S3: Nucleotide sequence alignment of *ALDH1A1* cloned from Liaoning cashmere goat and the *Capra hircus* reference sequence from NCBI GenBank; Figure S4: Uncropped Western blot images of ALDH1A1 protein expression in Liaoning cashmere goat skin across the seasonally assigned hair-cycle periods, corresponding to Figure 6C; Figure S5: Map of the pcDNA3.1(+)-*ALDH1A1* overexpression construct; Figure S6: Independent validation of proliferation-related responses using si-*ALDH1A1*-2 in SHFSCs; Figure S7: Representative uncropped Western blot images of ALDH1A1 protein expression in SHFSCs after *ALDH1A1* knockdown and overexpression, corresponding to Figure 8; Figure S8: Representative uncropped Western blot images of KRT19, integrin β1, and PCNA protein expression in SHFSCs after *ALDH1A1* knockdown and overexpression, corresponding to Figure 9G,H; Figure S9: Representative gating strategy for cell-cycle analysis of SHFSCs by flow cytometry; Figure S10: Original colony-formation source images; Figure S11: Representative bright-field images of primary SHFSCs; Data S1: SnapGene file of the pcDNA3.1(+)-*ALDH1A1* overexpression construct containing the complete plasmid sequence, feature annotations, and associated Sanger sequencing trace records; Data S2: Representative flow cytometry FCS files from one independent experimental run used for cell-cycle analysis of SHFSCs; Data S3: Retained blank-corrected CCK-8 OD450 measurements and corresponding statistical summaries for si-*ALDH1A1*-1 and si-*ALDH1A1*-2.

## Abbreviations

The following abbreviations are used in this manuscript:

ALDH1A1	Aldehyde dehydrogenase 1 family member A1
RA	Retinoic acid
ORF	Open reading frame
SHFSCs	Secondary hair follicle stem cells
ALDH-SF	Aldehyde dehydrogenase superfamily
HFSCs	Hair follicle stem cells
ALDH	Aldehyde dehydrogenase
FBS	Fetal bovine serum
CDD	Conserved Domain Database
siRNA	Small interfering RNA
qRT-PCR	Quantitative real-time PCR
CCK-8	Cell Counting Kit-8
EdU	5-Ethynyl-2′-deoxyuridine
DAPI	4′,6-Diamidino-2-phenylindole
PBS	Phosphate-buffered saline
BCA	Bicinchoninic acid
SDS-PAGE	Sodium dodecyl sulfate–polyacrylamide gel electrophoresis
PVDF	Polyvinylidene difluoride
TBST	Tris-buffered saline containing Tween 20
HRP	Horseradish peroxidase
ANOVA	Analysis of variance
HSD	Honestly significant difference
SHFs	Secondary hair follicles
DMSO	Dimethyl sulfoxide
OE-*ALDH1A1*	*ALDH1A1* overexpression
GAPDH	Glyceraldehyde-3-phosphate dehydrogenase
FSC-A	Forward-scatter area
SSC-A	Side-scatter area
SD	Standard deviation
PCR	Polymerase chain reaction
RAR	Retinoic acid receptor
PI3K/AKT	Phosphoinositide 3-kinase/Protein kinase B
PI-A	Propidium iodide area
PI-W	Propidium iodide width
GMQE	Global Model Quality Estimate
NAD	Nicotinamide adenine dinucleotide
FCS	Flow Cytometry Standard
