# Supplementary material for: Molecular Characterization of ALDH1A1 and Its Association with Secondary Hair Follicle Stem Cell Proliferation in Liaoning Cashmere Goats
Source: Cells. 2026 Oct 14;15(20):1865. doi: 10.3390/cells15201865 (PMC13702987; doi:10.3390/cells15201865)
Supplement: Supplementary file 1 [file cells-15-01865-s001.zip › Supplementary_Materials_ALDH1A1_2026.10.8 .pdf]

Supplementary Materials

for

Molecular Characterization of *ALDH1A1* and Its Association with Secondary Hair Follicle Stem Cell Proliferation in Liaoning Cashmere Goats

Table S1. Primer sequences used in this study.

Gene	NCBI Reference Sequence	Primer	Sequence (5'-3')	Primer length (bp)	Application
ALDH1A1	XM_005683753.3	Cloning-F	ATGTCGTCCTCAGCCATGCC	20	Gene cloning
ALDH1A1		Cloning-R	TTATGAGTTCTGAGAAATTTTGAT	24	Gene cloning
ALDH1A1	XM_005683753.3	qPCR-F	CATGGCGTATGGATGCTTCAG	21	qRT-PCR
ALDH1A1		qPCR-R	CCACCATTCATAGCCTCCATTGTC	24	qRT-PCR
KRT19	XM_018065060.1	qPCR-F	TGAGCGTGGAGGAGACTGACATC	23	qRT-PCR
KRT19		qPCR-R	TCGTGTGTGTTCTTCTTCAGGTAGG	25	qRT-PCR
ITGB1	XM_018056844.1	qPCR-F	TCAGTGCCGAGCCTTCAATA	20	qRT-PCR
ITGB1		qPCR-R	AGTAGAACCAGCAGTCGTCAA	21	qRT-PCR
PCNA	XM_005688167.3	qPCR-F	AGTGGCGTGAACCTACAGAGC	21	qRT-PCR
PCNA		qPCR-R	TACGGTCGCAGCGGTAAGTG	20	qRT-PCR
β-actin	NC_030832.1	qPCR-F	GGTGCCCATCTACGAGG	17	Reference gene
β-actin		qPCR-R	CTTGATGTCACGGAGATT	18	Reference gene

Table S2. Software, databases, and online resources used for sequence and structural analyses in this study.

Software/database	Application in this study	Access link
DNAMAN 9.0	Sequence assembly, alignment, and translation	https://www.lynnon.com/dnaman.html
MEGA 11	Phylogenetic analysis with 1000 bootstrap replicates	https://www.megasoftware.net/
ExPASy ProtParam	Physicochemical properties, molecular weight, and amino acid composition	https://web.expasy.org/protparam/
SignalP 6.0	Signal peptide prediction	https://services.healthtech.dtu.dk/services/SignalP-6.0/
TMHMM 2.0	Transmembrane-helix prediction	https://services.healthtech.dtu.dk/services/TMHMM-2.0/
NetPhos 3.1	Prediction of potential phosphorylation sites	https://services.healthtech.dtu.dk/services/NetPhos-3.1/
NCBI Conserved Domain Database (CDD)	Conserved-domain analysis	https://www.ncbi.nlm.nih.gov/Structure/cdd/wrpsb.cgi
SOPMA	Secondary-structure prediction	https://npsa-prabi.ibcp.fr/cgibin/npsa_automat.pl?page=/NPSA/npsa_sopma_f.html
SWISS-MODEL	Homology modeling	https://swissmodel.expasy.org/
ProSA-web	Protein-model quality evaluation	https://prosa.services.came.sbg.ac.at/prosa.php
PyMOL 3.0	Three-dimensional structure visualization and annotation	https://pymol.org/
MolProbity v4.4	Protein-model quality evaluation	http://molprobity.biochem.duke.edu

Table S3. siRNA sequences used for *ALDH1A1* knockdown.

siRNA	Sense Strand (5'-3')	Antisense Strand (5'-3')	Final Concentration (nM)	Used in Functional Assays
si-*ALDH1A1*-1	GAGUGGUAAGAAAUUUCCAGUCUUU	AAAGACUGGAAAUUUCUUACCACUC	100	Yes, main functional assays
si-*ALDH1A1*-2	GACUGCUCUUCACAUGGGAUCUUUA	UAAAGAUCCCAUGUGAAGAGCAGUC	100	Yes, independent CCK-8 and EdU validation
si-*ALDH1A1*-3	GGAACCGUGUGGGUGAACUGCUAUA	UAUAGCAGUUCACCCACACGGUUCC	100	No
si-control	UUCUCCGAACGUGUCACGUTT	ACGUGACACGUUCGGAGAATT	100	Control

Table S4. Primary and secondary antibodies used in this study.

Target	Host Species	Manufacturer	Catalog No.	Dilution	Expected MW (kDa)
ALDH1A1	rabbit	Abmart	PC10162S	1:1000	54
KRT19	rabbit	ABclonal	A0247	1:1000	44
KRT15	rabbit	ABclonal	A19030	1:1000	49
Integrin β1	rabbit	ABclonal	A2217	1:500	135
PCNA	rabbit	ABclonal	A0264SP	1:1000	34
GAPDH	rabbit	ABclonal	A19056	1:20000	36
Anti-rabbit IgG-HRP	Donkey	ABclonal	AS038	1:20000	N/A
ABflo® 488-conjugated Goat anti-Rabbit	Goat	ABclonal	A24925	1:200	N/A
ABflo® 647-conjugated Goat anti-Rabbit IgG	Goat	ABclonal	AS060	1:200	N/A

Figure S1. PCR amplification of the full-length *ALDH1A1* coding sequence from Liaoning cashmere goat skin cDNA.

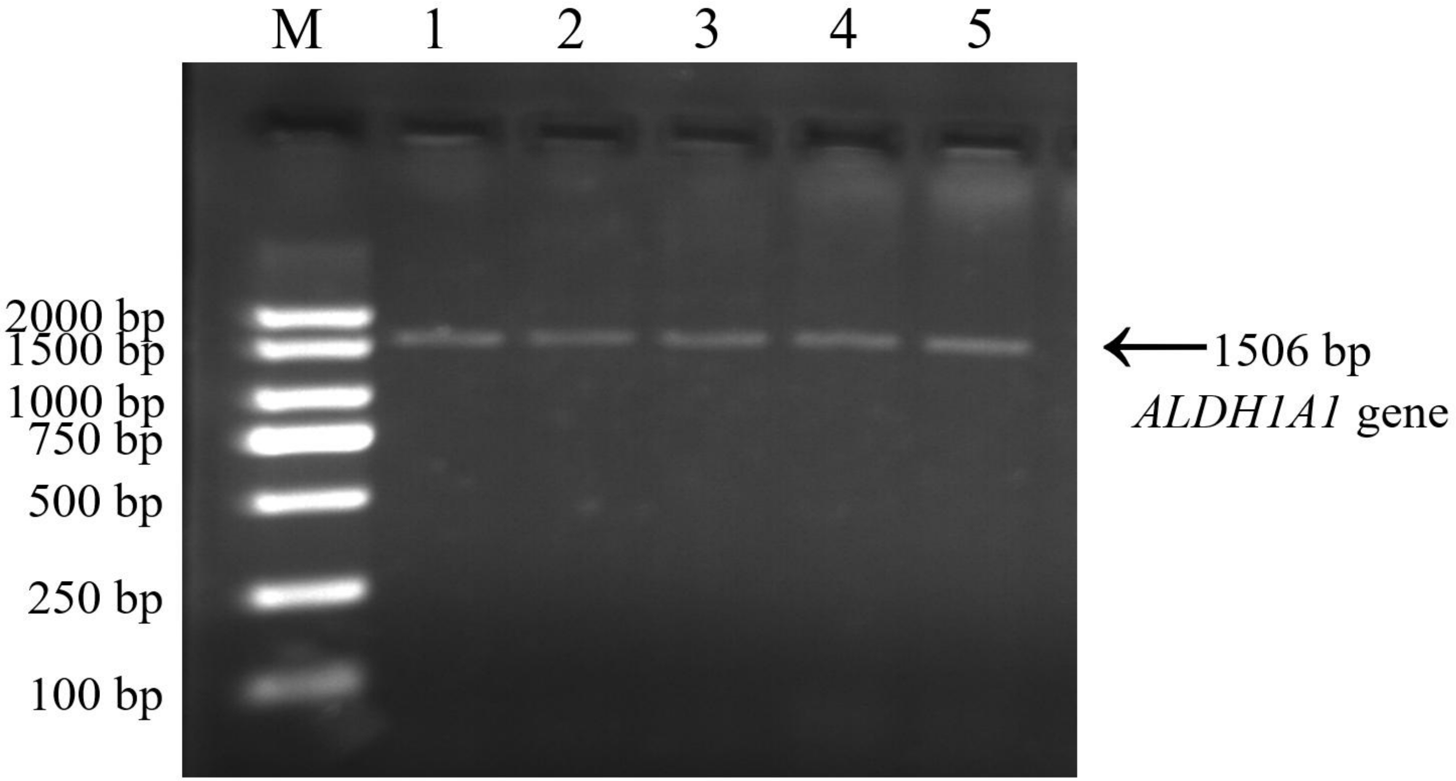

M, DNA marker; lanes 1–5, *ALDH1A1* PCR products. The expected amplicon size is 1506 bp.

Figure S2. In silico prediction of the Liaoning cashmere goat ALDH1A1 protein.

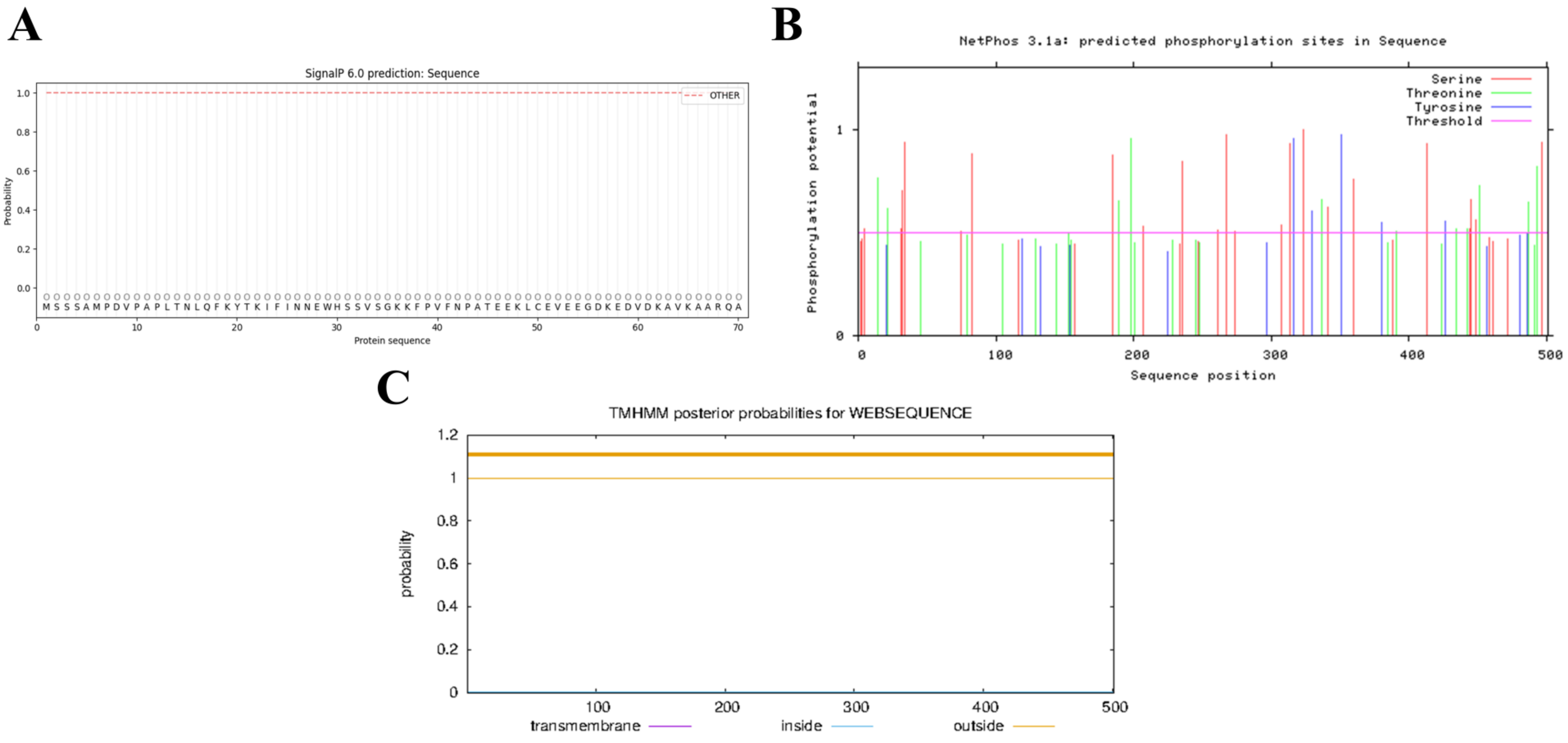

(**A**) Signal peptide prediction by SignalP 6.0. (**B**) Phosphorylation site prediction by NetPhos 3.1. (**C**) Transmembrane helix prediction by TMHMM 2.0.

Figure S3. Nucleotide sequence alignment of *ALDH1A1* cloned from Liaoning cashmere goat and the Capra hircus reference sequence from NCBI GenBank.

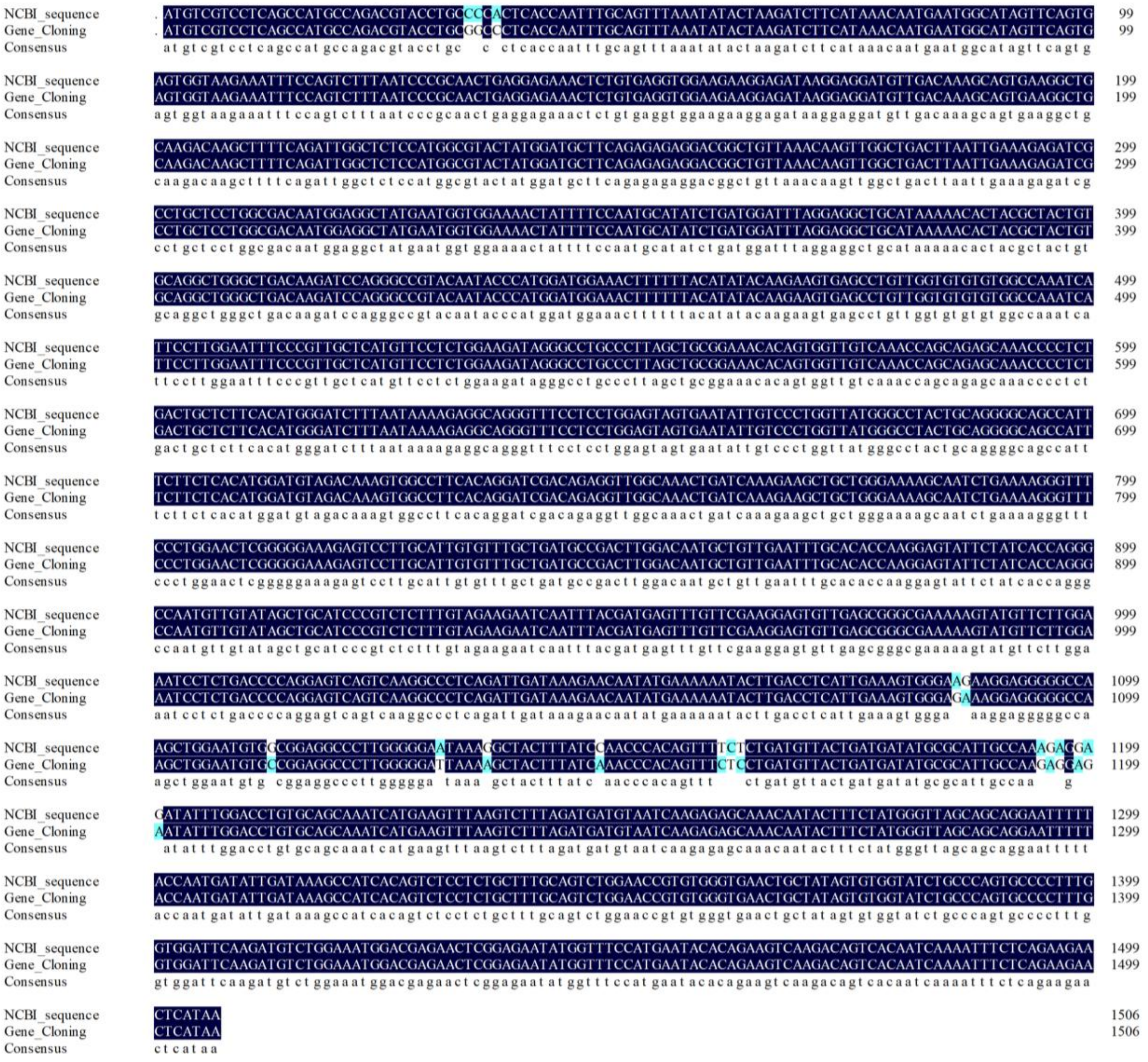

The top sequence is the standard *ALDH1A1* reference sequence of domestic goat (*Capra hircus*) retrieved from NCBI; the bottom sequence is the full-length coding sequence of *ALDH1A1* obtained by gene cloning from Liaoning cashmere goat in the present study. Divergent nucleotide sites between the two sequences are highlighted with a light blue background. The numbers on the right indicate nucleotide positions.

Figure S4. Uncropped Western blot images of ALDH1A1 protein expression in Liaoning cashmere goat skin across the seasonally assigned hair-cycle periods, corresponding to Figure 6C.

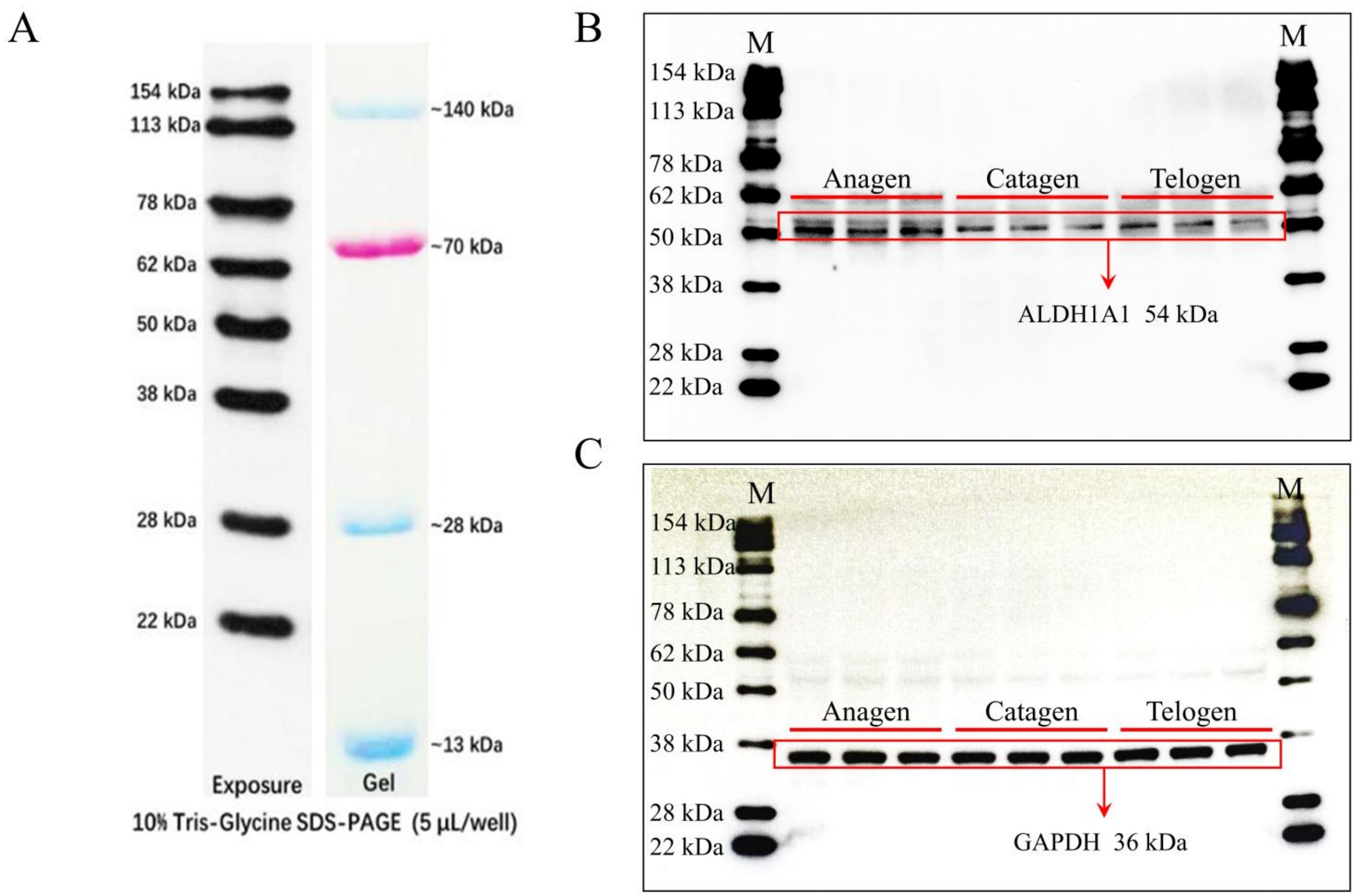

(**A**) Prestained protein molecular weight marker standard profile; the left column shows the chemiluminescence-exposed marker bands, and the right column shows the stained gel bands (Biosharp, catalog No. BL1106A); (**B**) ALDH1A1 (54 kDa) protein bands; (**C**) GAPDH (36 kDa) protein bands served as the loading control. M indicates molecular weight markers, with molecular weights (kDa) indicated on the left or right of each membrane. Red lines denote the lanes: anagen, catagen, and telogen. Red boxes indicate the cropped regions shown in the main figure (Figure 6C). Full-length blots are presented in this supplementary figure.

Figure S5. Map of the pcDNA3.1(+)-*ALDH1A1* overexpression construct. The recombinant plasmid contains the complete 1506-bp ALDH1A1 coding sequence inserted into the pcDNA3.1(+) vector through the KpnI and EcoRI restriction sites.

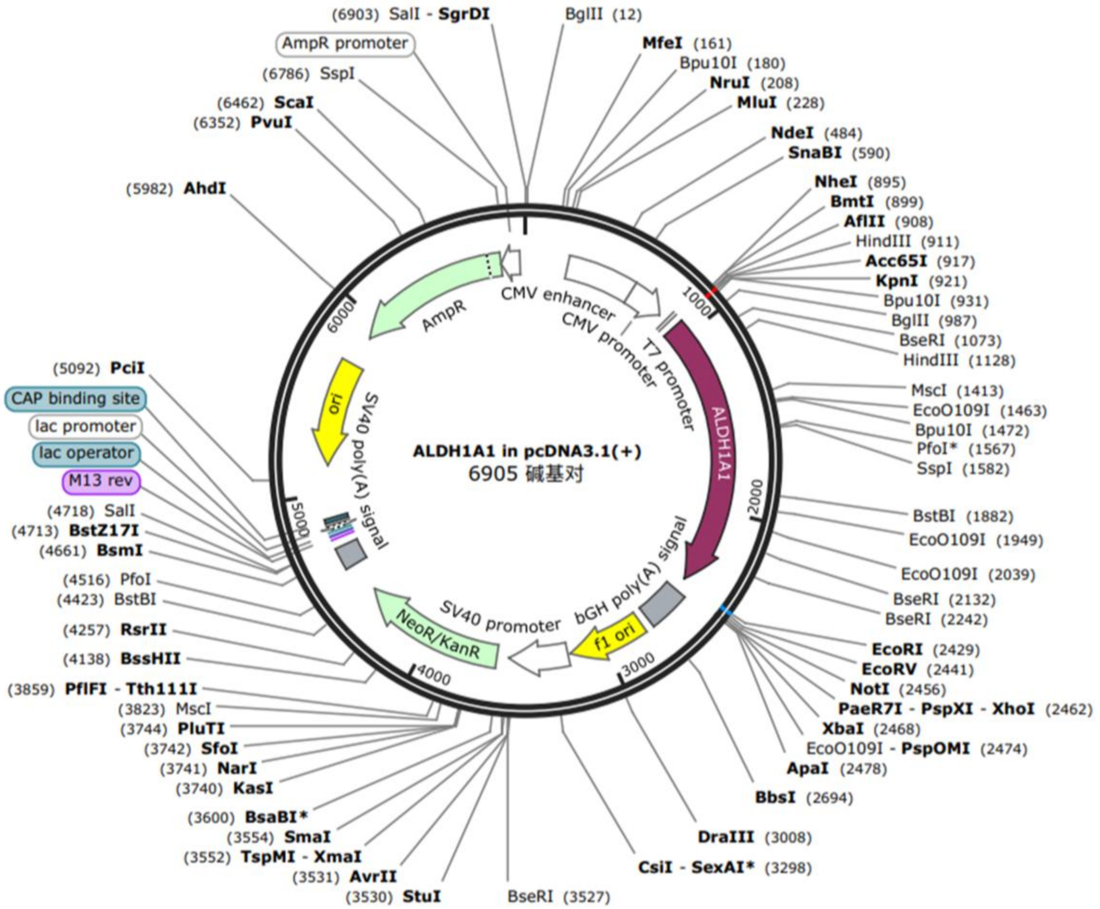

Figure S6. Independent validation of proliferation-related responses using si-*ALDH1A1*-2 in SHFSCs.

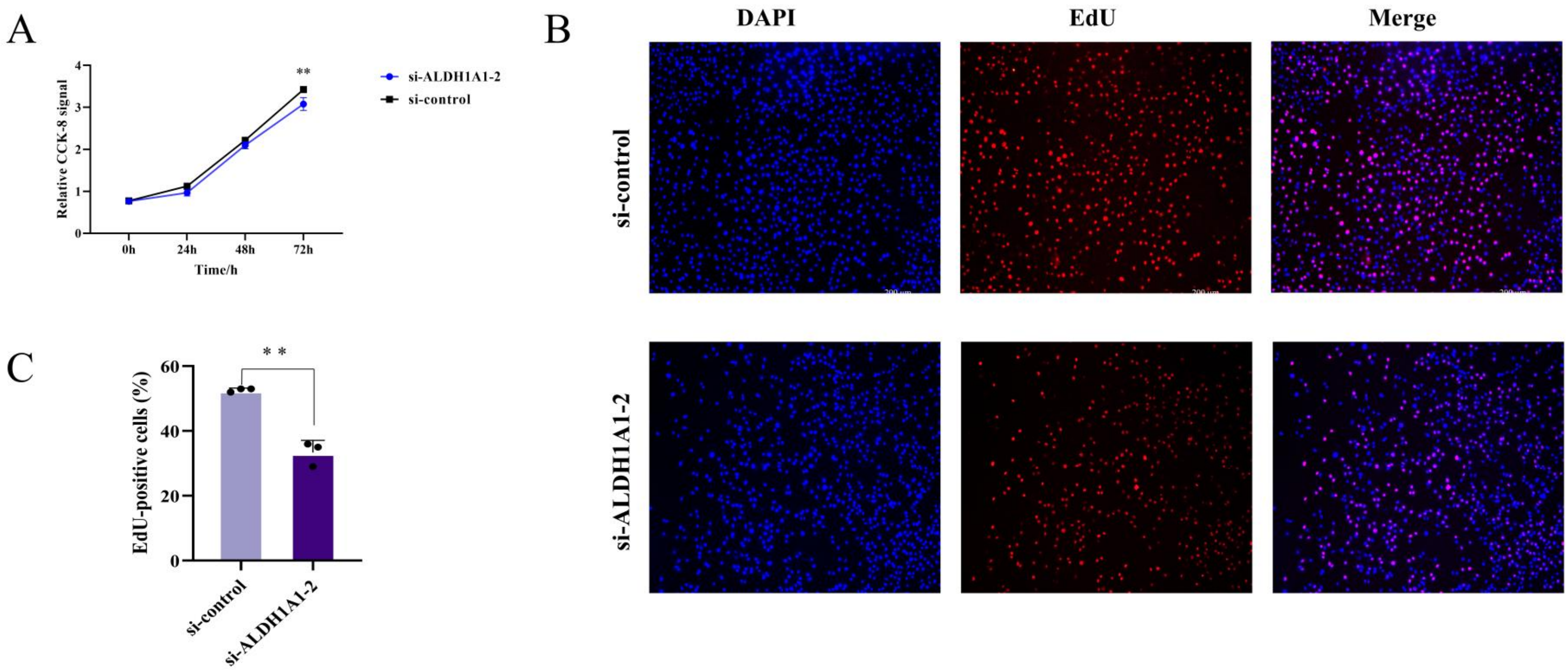

(**A**) Time-dependent CCK-8 signals in the si-control and si-*ALDH1A1*-2 groups. (**B**) Representative EdU staining images. Blue, DAPI-stained nuclei; red, EdU-positive cells; Merge, merged channels. (**C**) Quantification of the EdU-positive cell fraction. Data are shown as mean ± SD from three independent experimental repeats using primary SHFSCs derived from a single donor goat (donor n = 1). Technical replicates: CCK-8, five wells per treatment/time point; EdU, three wells per treatment. Statistical analysis: linear mixed-effects model with Šídák-adjusted comparisons for CCK-8 and paired t-test for EdU. *$p < 0.05$; **$p < 0.01$.

Figure S7. Representative uncropped Western blot images of ALDH1A1 protein expression in SHFSCs after ALDH1A1 knockdown and overexpression, corresponding to Figure 8

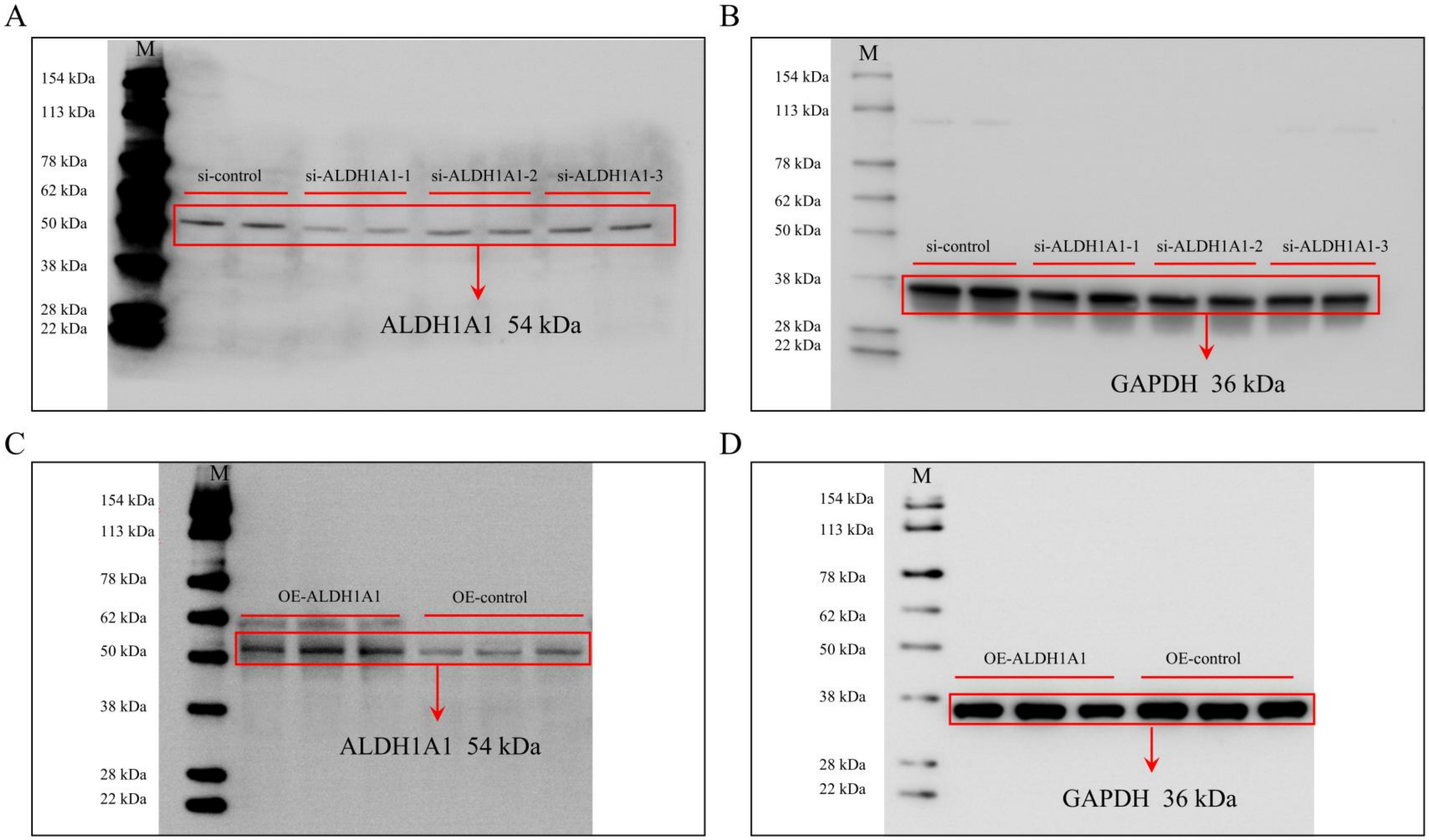

(**A,B**) ALDH1A1 (54 kDa) and GAPDH (36 kDa) protein bands after *ALDH1A1* knockdown; (**C,D**) ALDH1A1 (54 kDa) and GAPDH (36 kDa) protein bands after *ALDH1A1* overexpression. M indicates molecular weight markers, with molecular weights (kDa) indicated on the left of each membrane. Red lines denote the lanes for si-control, si-*ALDH1A1*-1, si-*ALDH1A1*-2, si-*ALDH1A1*-3, the overexpression control (OE-control), and *ALDH1A1* overexpression (OE-*ALDH1A1*). Full-length blots are presented in this supplementary figure.

Figure S8. Representative uncropped Western blot images of KRT19, integrin β1, and PCNA protein expression in SHFSCs after ALDH1A1 knockdown and overexpression, corresponding to Figure 9G and H.

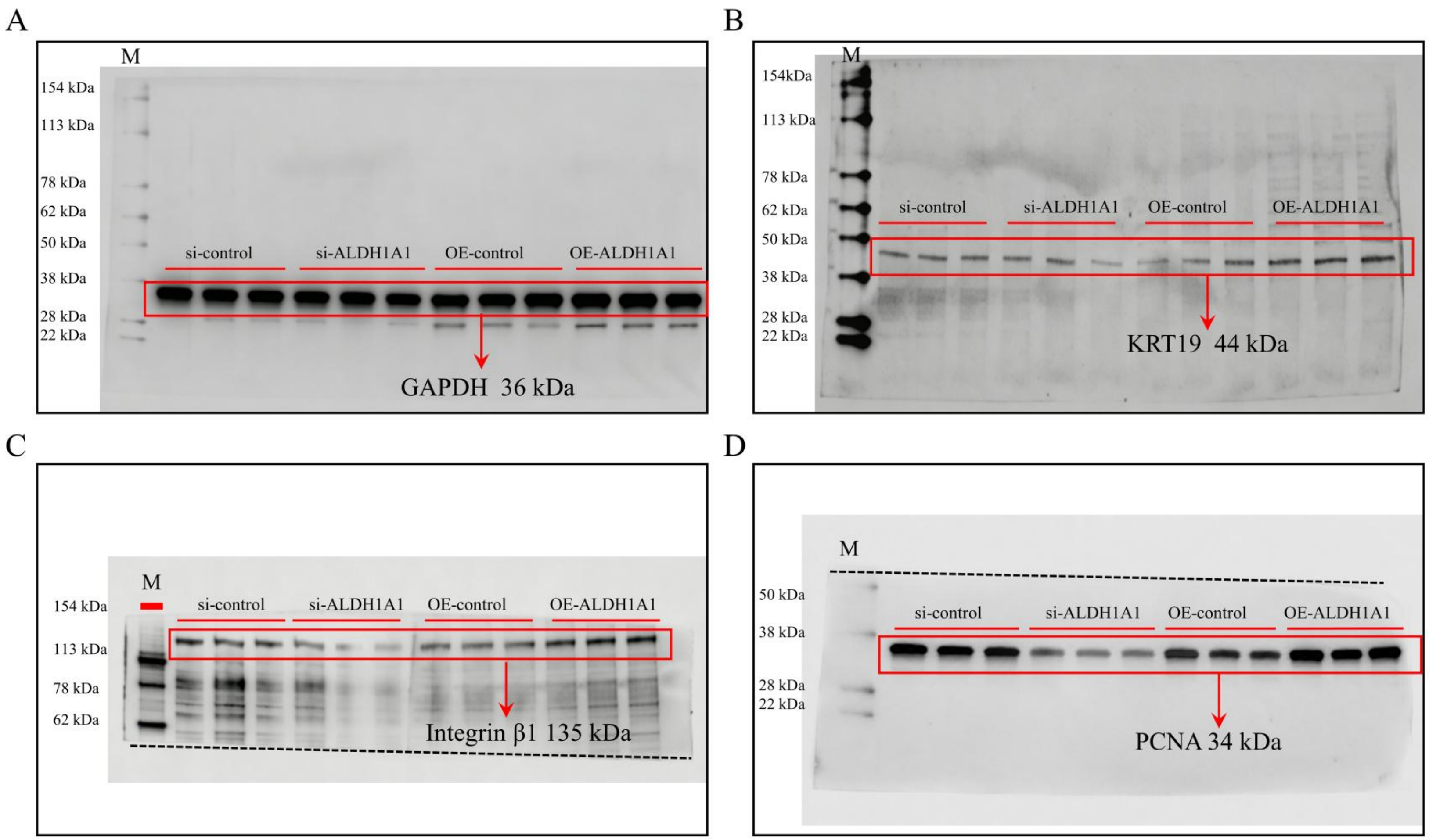

(**A**) GAPDH (36 kDa) protein bands served as the loading control; (**B**) KRT19 (44 kDa) protein bands; (**C**) Integrin β1 (135 kDa) protein bands; (**D**) PCNA (34 kDa) protein bands. M indicates molecular weight markers, with molecular weights (kDa) indicated on the left of each membrane. Red lines denote the lanes for si-control, si-*ALDH1A1*, the overexpression control (OE-control), and *ALDH1A1* overexpression (OE-*ALDH1A1*). For (C) and (D), membranes were cut along the black dashed lines prior to antibody incubation according to the molecular weights of target proteins to minimize antibody consumption. Red boxes indicate the cropped regions shown in the main figure (Figure 9G and H). Full-length blots are presented in this supplementary figure.

Figure S9. Representative gating strategy for cell-cycle analysis of SHFSCs by flow cytometry

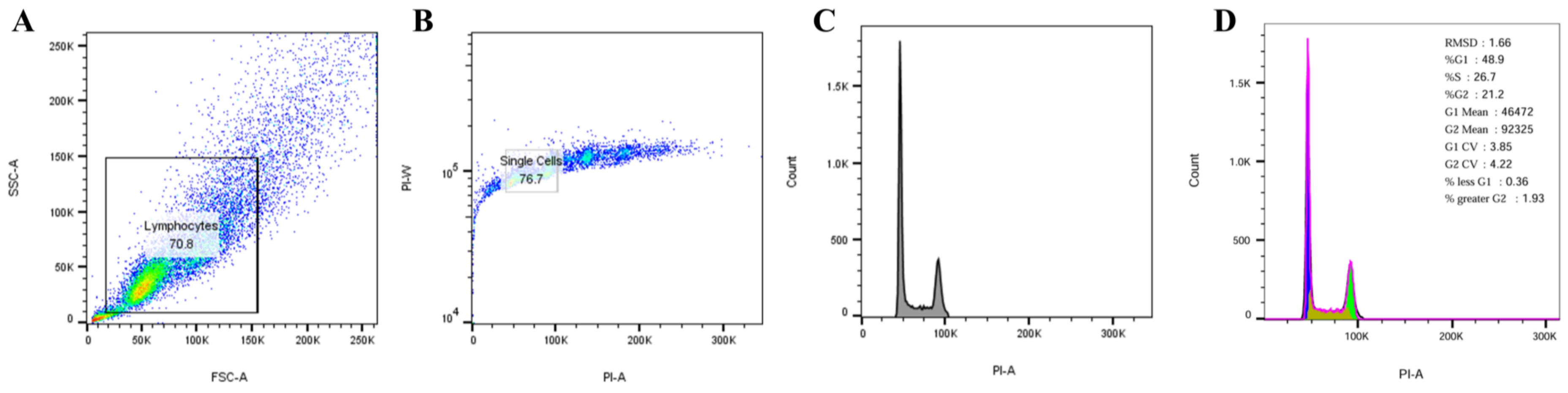

(**A**) The main cell population was identified on FSC-A versus SSC-A plots to exclude cellular debris. (**B**) Single cells were selected using PI-A versus PI-W plots to exclude doublets and cell aggregates. (**C**) Representative PI-A DNA-content histogram of gated singlet cells. (**D**) Representative cell-cycle fitting using the Watson pragmatic model for quantification of G0/G1, S, and G2/M phase distributions.

Figure S10. Original colony-formation source images.

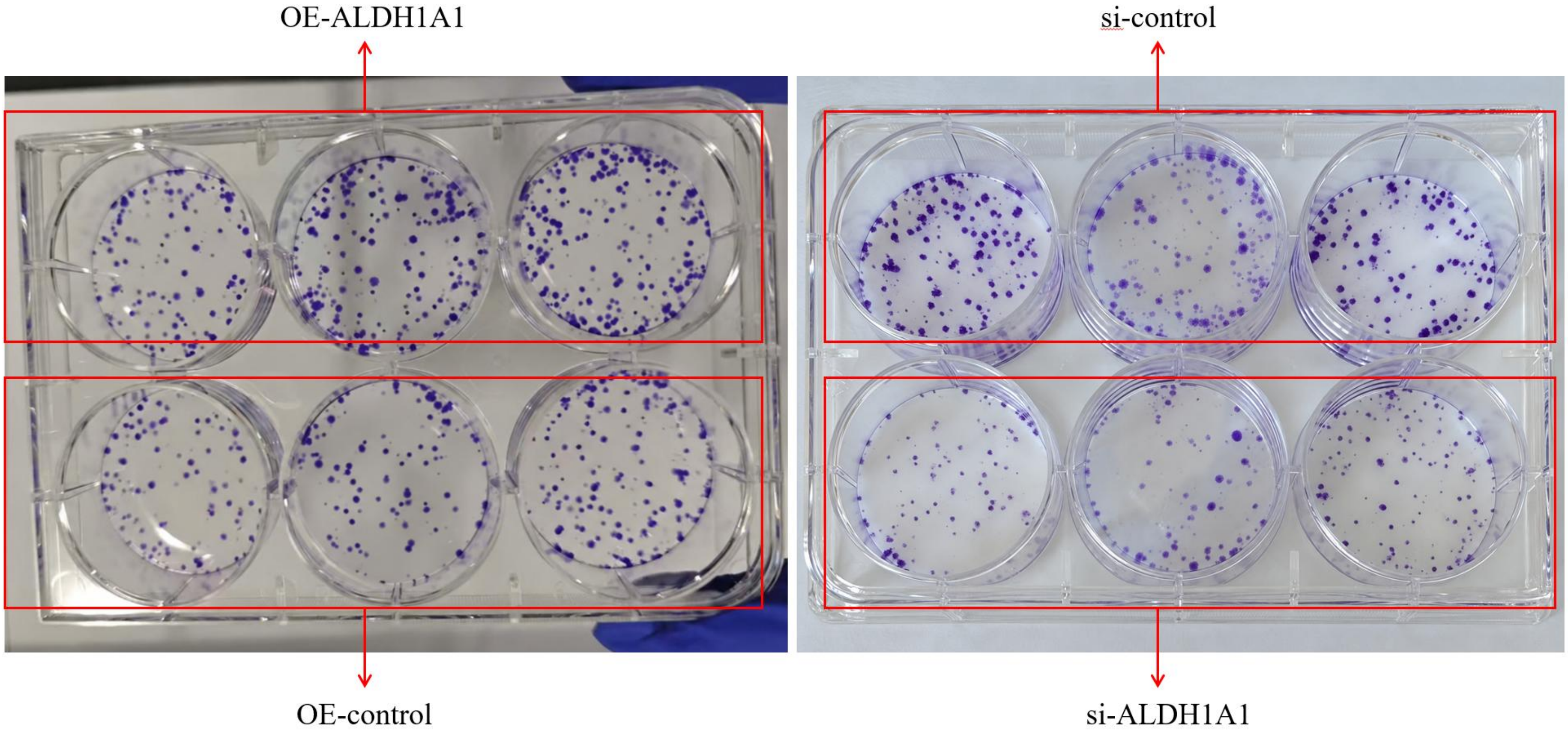

Figure S11. Representative bright-field images of primary SHFSCs

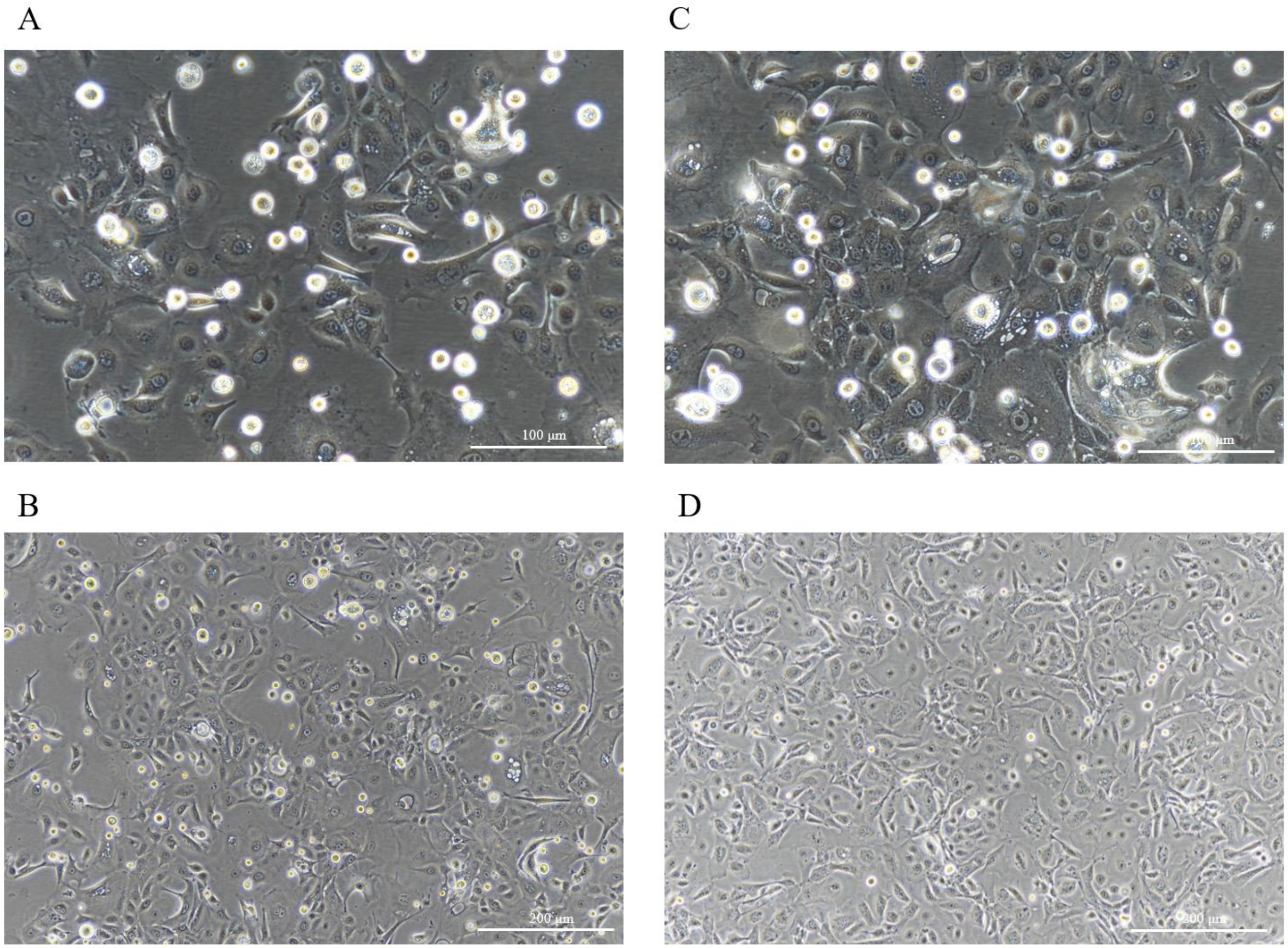

(**A, B**) Representative bright-field images of primary SHFSCs at the early primary culture stage, captured at 200× (A) and 100× (B) magnification. (**C, D**) Representative brightfield images of passaged SHFSCs, captured at 200× (C) and 100× (D) magnification. Scale bars: 100 μm (200×, A and C); 200 μm (100×, B and D).
